# Loss of kinesin-8 improves the robustness of the self-assembled spindle in *Schizosaccharomyces pombe*

**DOI:** 10.1242/jcs.253799

**Published:** 2021-08-23

**Authors:** Alberto Pineda-Santaella, Nazaret Fernández-Castillo, Alberto Jiménez-Martín, María del Carmen Macías-Cabeza, Ángela Sánchez-Gómez, Alfonso Fernández-Álvarez

**Affiliations:** Andalusian Centre for Developmental Biology (CABD), Universidad Pablo de Olavide – Consejo Superior de Investigaciones Científicas (CSIC), Junta de Andalucía, Ctra. Utrera Km. 4, 41013 Seville, Spain

**Keywords:** Meiosis, Spindle pole body, Acentrosomal spindle, *Schizosaccharomyces pombe*

## Abstract

Chromosome segregation in female meiosis in many metazoans is mediated by acentrosomal spindles, the existence of which implies that microtubule spindles self-assemble without the participation of the centrosomes. Although it is thought that acentrosomal meiosis is not conserved in fungi, we recently reported the formation of self-assembled microtubule arrays, which were able to segregate chromosomes, in fission yeast mutants, in which the contribution of the spindle pole body (SPB; the centrosome equivalent in yeast) was specifically blocked during meiosis. Here, we demonstrate that this unexpected microtubule formation represents a bona fide type of acentrosomal spindle. Moreover, a comparative analysis of these self-assembled spindles and the canonical SPB-dependent spindle reveals similarities and differences; for example, both spindles have a similar polarity, but the location of the γ-tubulin complex differs. We also show that the robustness of self-assembled spindles can be reinforced by eliminating kinesin-8 family members, whereas kinesin-8 mutants have an adverse impact on SPB-dependent spindles. Hence, we consider that reinforced self-assembled spindles in yeast will help to clarify the molecular mechanisms behind acentrosomal meiosis, a crucial step towards better understanding gametogenesis.

## Abstract (precis)

**Summary:** We report a comparative analysis of self-assembled spindles and canonical centrosomal spindles in fission yeast, which could clarify the mechanisms underlying acentrosomal meiosis.

## INTRODUCTION

Meiosis is a specialized type of cell division in which a diploid progenitor cell undergoes two reductional divisions, namely meiosis I (MI) and meiosis II (MII), to produce haploid cells (Griswold and Hunt, 2013). Chromosome segregation during meiotic divisions is mediated by the spindle, which consists of microtubules together with a vast cohort of structural and regulatory proteins. In mitosis and male meiosis, spindle formation is mediated by the centrosomes, the major microtubule-organizing centres in the cell, which localize to the spindle poles (Walczak and Heald, 2008). Centrosomes contain two microtubule-derived structures called centrioles, which are surrounded by the pericentriolar material, a proteinaceous electron-dense matrix (Bettencourt-Dias and Glover, 2007).

By contrast, in female meiosis, centrosomes are purposefully eliminated in oocytes, the gamete progenitor cells, before or during the meiotic divisions (Dumont and Desai, 2012). Generally, oocytes specifically degrade centrioles while maintaining the pericentriolar material (Schatten, 1994). Early eukaryotic species of echinoderms (Nakashima and Kato, 2001; Sluder et al., 1993), bivalvia (Longo and Anderson, 1969) and crustaceans (Ruthmann, 1959) enter meiosis with four centrioles (two centrosomes) and gradually expel them to the degenerating daughter cells in each meiotic division. This leaves the mature oocyte with one centriole, which ends up disintegrating. Late eukaryotes degrade centrosomes in earlier meiotic stages, using a wide range of elimination processes. *Drosophila melanogaster* oocytes initially possess a multicentriolar microtubule-organizing centre, but this is fully degraded before the onset of meiotic divisions (Colombié et al., 2008; Cooley and Theurkauf, 1994; Gonzalez et al., 1998; Theurkauf and Hawley, 1992).

Similarly, in *Caenorhabditis elegans* oocytes (Kemp et al., 2004; Srayko et al., 2006; Wolff et al., 2016), *Xenopus* extracts (Gard, 1991; Heald et al., 1996; Walczak et al., 1998) and human oocytes, centrioles are lost throughout pre-meiotic phases (Hertig and Adams, 1967; Sathananthan et al., 2006). As a consequence of centriole degradation, the meiotic spindle in these late eukaryotic species must form and segregate chromosomes in the absence of complete, functional centrosomes; this is why this particular type of spindle is known as an acentrosomal spindle. Mouse oocytes are an exception, because they substitute centrosomes with multiple acentriolar microtubule-organizing centres (Szollosi et al., 1972) that eventually collapse to form the meiotic spindle poles (Clift and Schuh, 2015; Kolano et al., 2012; Schuh and Ellenberg, 2007). The common absence of centrosomes in female animal meiosis leads to error-prone chromosome segregation that is mediated by acentrosomal spindles (Holubcova et al., 2015). The molecular basis of this process is not well understood, owing in part to the low availability of oocytes for scientific research, especially from mammals. In this work, we use the fission yeast *Schizosaccharomyces pombe* to explore some of the mechanisms behind self-assembled spindle formation during gametogenesis.

Mitotic and meiotic spindles are nucleated in *S. pombe* by the spindle pole bodies (SPBs; the centrosome equivalent in yeast). During interphase, one SPB sits on the cytoplasmic side of the nuclear envelope (NE). Once the mitotic or meiotic cell cycle is initiated, it duplicates into two SPBs, which are then inserted into the partially disassembled portion of the NE underneath them (Bestul et al., 2017; Ding et al., 1997; McCully and Robinow, 1971; Tanaka and Kanbe, 1986), while the rest of the NE remains intact (Yoshida and Sazer, 2004). This insertion allows SPBs to access the nucleoplasm and start to organize nuclear microtubules into a spindle, which eventually segregates chromosomes. Localized NE disassembly and SPB insertion are controlled by the interaction of specialized regions of chromosomes, centromeres (in mitosis) or telomeres (in meiosis), and the linker of nucleoskeleton and cytoskeleton (LINC) complex (Fennell et al., 2015; Fernández-Álvarez et al., 2016; Tomita and Cooper, 2007). The LINC complex is composed of two groups of proteins that link centromeres and telomeres with the SPBs: SUN-domain proteins, which span the inner nuclear membrane; and KASH-domain proteins, which span the outer nuclear membrane (Rothballer et al., 2013).

In particular, during meiotic prophase, telomeres are gathered beneath the SPB in a chromosomal arrangement called the telomere bouquet (Niwa et al., 2000; Robinow, 1977), which bridges them via the telomeric proteins Taz1 and Rap1 and the meiosis-specific Bqt1–Bqt2 dimer to Sad1, the SUN-domain protein in fission yeast (Chikashige et al., 2006). Disruption of telomere–Sad1 interaction, e.g. by deletion of *bqt1*, abolishes bouquet formation, localized NE disassembly and insertion of SPBs, which remain uninserted and distant from the NE. Consequently, the absence of the bouquet abolishes SPB-mediated spindle formation (Tomita and Cooper, 2007). Centromeres have the ability to substitute for telomeres, rescuing meiotic SPB insertion into the NE and spindle formation in bouquet-deficient cells, owing to their residual interaction with Sad1 (Fennell et al., 2015). A combined double point mutation in *sad1*, *sad1.2* (*sad1.T3S.S52P*), impairs centromere–Sad1 interaction in meiotic prophase, which renders ∼100% of *bqt1*Δ *sad1*.*2* cells unable to insert the SPBs into the NE and form an SPB-mediated spindle (Fernández-Álvarez et al., 2016; Pineda-Santaella and Fernández-Álvarez, 2019). In this *bqt1*Δ *sad1*.*2* background, we recently observed the formation of a nuclear microtubule array structure, which was able to segregate chromosomes, in ∼60–80% of the cells (Pineda-Santaella and Fernández-Álvarez, 2019). This finding prompted us to hypothesize that this structure might be a type of self-assembled spindle similar to that found in metazoan acentrosomal meiosis. In this study, we establish that the microtubule organization observed in the absence of SPB insertion is a bona fide self-assembled spindle. We describe the molecular characterization of this type of acentrosomal spindle in fission yeast, with the aim of understanding the molecular basis of acentrosomal spindle structure and function.

## RESULTS

### Self-assembled spindle formation and polarization are independent of the LINC complex

**Fig. 1.**
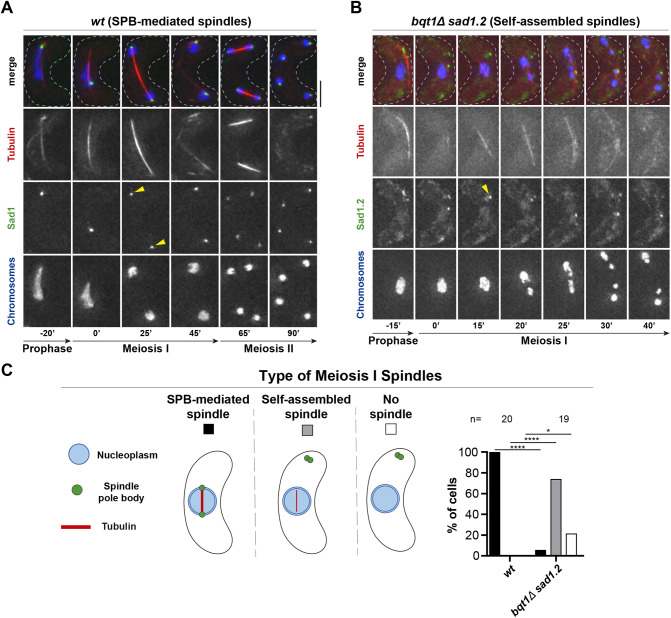
**LINC complex does not localize at self-assembled spindle poles.** (A,B) Frames from films of meiocytes carrying mCherry–Atb2 (ectopically expressed, tubulin) and Hht1–CFP (at one of the two endogenous *hht1^+^* loci). Endogenously tagged Sad1–GFP (A) and Sad1.2–GFP (B), LINC. Numbering indicates meiotic progression in minutes; *t*=0 is just before spindle formation. Scale bar: 5 µm. Yellow arrowheads indicate the location of Sad1–GFP or Sad1.2–GFP during spindle pole body (SPB)-mediated spindle formation and self-assembled spindles. The phenotype shown in B, Sad1.2 does not localize at self-assembled spindle poles, is observed in 100% of *bqt1*Δ *sad1.2* meiocytes (19 cells were scored from more than two independent experiments). All wild-type (*wt*) cells showed Sad1–GFP at spindle poles (*n*=20). (C) Comparison of type of meiotic spindle in the *wt* and *bqt1*Δ *sad1.2* backgrounds shown in A and B. The percentage of self-assembled spindle formation was consistent with previous observations (Pineda-Santaella and Fernández-Álvarez, 2019). Significant differences from *wt* calculated using Fisher's exact test: ****P<0.0001; *P<0.05. *n* cells were scored from more than two independent experiments.

Previous studies using electron microscopy have shown that the full SPB structure in bouquet-defective cells (*bqt1*Δ) fails to insert into the NE and is displaced into the cytoplasm (Fernández-Álvarez et al., 2016). This phenotype, together with the fact that the formation of microtubule arrangements occurs in the nuclear environment while the SPB localizes far from the nucleus (Pineda-Santaella and Fernández-Álvarez, 2019), strongly suggests that the array of microtubules observed in *bqt1*Δ *sad1*.*2* meiocytes is assembled without the participation of the SPB. To further confirm the self-assembly of the microtubule array, we explored whether the LINC complex, which is permanently associated with the inner part of the SPB in fission yeast mitosis and meiosis at the NE (Hagan and Yanagida, 1995; Rothballer et al., 2013), might contribute to the formation of the microtubule array. In particular, we studied Sad1, the fission yeast SUN-domain protein, because it is the most internal part of the LINC complex facing the nucleoplasmic environment (Bestul et al., 2017). We endogenously tagged *sad1* and the allele *sad1.2* to visualize via live fluorescence microscopy their location in the absence of SPB insertion (*bqt1*Δ *sad1.2*) in meiosis. First, we compared the behaviour of Sad1–GFP and Sad1.2–GFP throughout meiosis in *bqt1^+^* cells (showing SPB-mediated spindles). As previously observed for mitotic cells (Fernández-Álvarez et al., 2016), *sad1.2* mutation did not alter Sad1 protein location at the SPBs in meiosis (Fig. 1A, 25′ and Fig. S1A, 30′, yellow arrowheads). Analysis of *bqt1*Δ *sad1.2-GFP* meiosis showed the formation of an array of polarized microtubules that were organized around chromosomes (self-assembled spindles) and had the capability to segregate them (Fig. 1B, 15′ to 40′; quantification in Fig. 1C). Unlike in the SPB-mediated spindles, Sad1.2 did not localize to the tips of the microtubule array (compare Fig. 1A, 25′ and B, 15′, yellow arrowheads), indicating that polarization of the microtubule array is independent of the location of the SUN-domain protein. These observations, together with our previous data (Pineda-Santaella and Fernández-Álvarez, 2019), indicate that the microtubule array in *bqt1*Δ *sad1.2* meiocytes is a type of functional self-assembled spindle, which is able to segregate chromosomes with kinetochores (Fig. S1B), the formation and polarization of which are not mediated by the localization of either the LINC complex or the SPB.

### Ase1/PRC1 is an essential structural component of meiotic self-assembled spindles

**Fig. 2.**
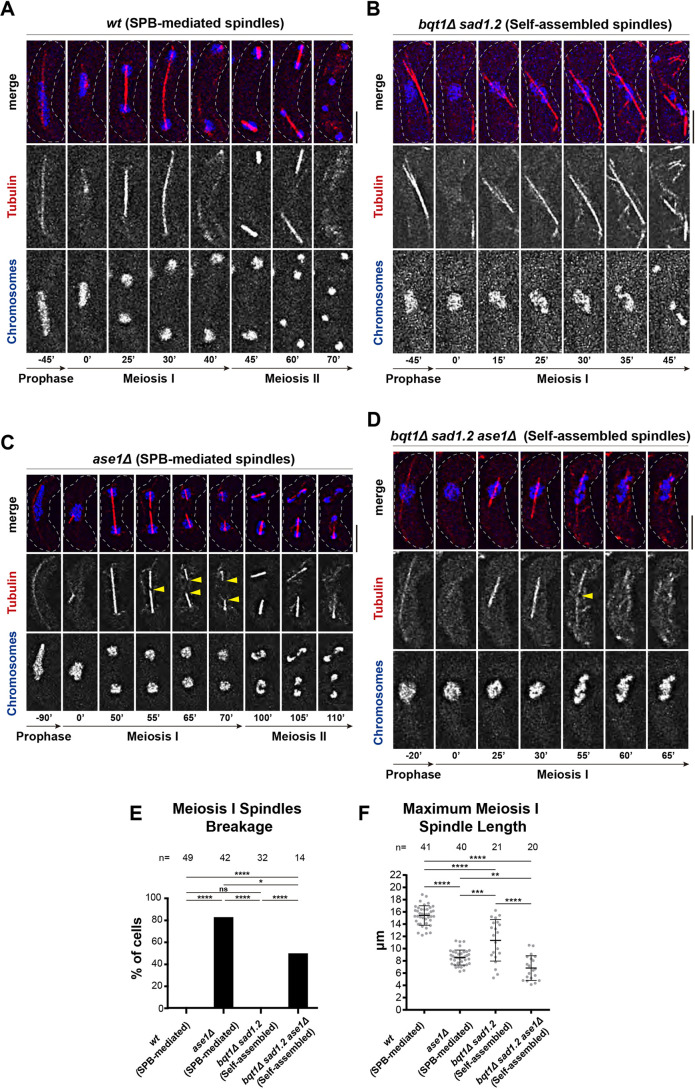
**Microtubule crosslinker Ase1/PRC1 is an essential component of self-assembled spindles.** (A–D) Frames from films of meiocytes of the indicated genotypes carrying chromosomes and spindles tagged as in Fig. 1. Numbering indicates meiotic progression in minutes; *t*=0 is just before spindle formation. Scale bars: 5 µm. (C) Yellow arrowheads indicate discrete breakage at the spindle structure (55′) and shrinkage of resulting spindle halves (65′ to 70′). (D) Yellow arrowhead indicates discrete breakage of the spindle structure (55′). (E) Quantification of MI spindle breakage in *ase1*^+^ and *ase1*Δ SPB-dependent and self-assembled spindles. Fisher's exact test: ****P<0.0001; *P<0.05; ns, *P*>0.05. (F) Maximum MI spindle length is quantified in SPB-mediated and self-assembled spindles. Fisher's exact test: ****P<0.0001; ***P<0.001, **<0.01. *n* is the total number of cells scored from more than three independent experiments. Bars represent mean and s.d.

To identify the main motor proteins that support the self-assembled spindles in *bqt1*Δ *sad1.2* meiocytes, we hypothesized that the major molecular mechanisms underlying SPB-dependent spindle formation might also be important for spindle self-assembly in bouquet-defective cells. We previously observed that the microtubule crosslinker protein Ase1/PRC1 localizes to the body of self-assembled spindles in very similar patterns to those seen for SPB-mediated spindles, including at the spindle midzone (Pineda-Santaella and Fernández-Álvarez, 2019). The location of Ase1 at the midzone in self-assembled spindles suggests that this protein might be part of the structure and might be involved in its integrity and extension. To uncover the functional relevance of Ase1 for self-assembled spindle behaviour, we analysed the effect of *ase1* deletion in meiosis. Stages of *ase1^+^* SPB-mediated spindles comprise nucleation (Fig. 2A, 0′), assembly (Fig. 2A, 0′ to 25′), elongation (Fig. 2A, 25′ to 30′) and disassembly, in which the whole spindle structure is dismantled (Fig. 2A, 30′ to 40′). We consistently observed similar stages for *ase1^+^* self-assembled spindles, comprising formation (Fig. 2B, 0′ to 15′), elongation (Fig. 2B, 15′ to 35′) and disassembly (Fig. 2B, 35′ to 45′). *ase1*Δ MI SPB-mediated spindles exhibited normal nucleation and assembly (Fig. 2C, 0′ to 50′), but, in contrast to *ase1^+^* cells, *ase1*Δ cells showed a discrete breakage specifically at the spindle midzone during elongation (Fig. 2C, 55′, yellow arrowhead; quantification in Fig. 2E); spindle microtubules shrank from the midzone, leading to spindle dissolution instead of global disassembly (Fig. 2C, 55′ to 70′, yellow arrowheads). Consistent with the role of Ase1 in spindle extension, the loss of Ase1 led to reduced spindle length (Fig. 2F) (Zheng et al., 2020). Strikingly, *ase1*Δ self-assembled spindles displayed a behaviour similar to that of *ase1*Δ SPB-mediated spindles, showing a local breakage at the spindle midzone instead of general disassembly (Fig. 2D, 55′ to 65′ and Fig. S2, 25′, yellow arrowheads; quantification in Fig. 2E). In addition, loss of Ase1 reduced the maximum spindle length in SPB-independent spindles (Fig. 2D and Fig. S2; quantification in Fig. 2F). These results establish that Ase1/PRC1 is essential for maintaining the structural integrity of self-assembled spindles in *bqt1*Δ *sad1.2* meiosis.

### Self-assembled spindles and SPB-mediated spindles share a similar polarity

We have shown that self-assembled spindles are organized around chromosomes and grow via bipolar extension that is not determined by the SPB or the LINC complex; for this reason, we wanted to establish whether these spindles are characterized by normal or inverted polarity. The fact that Ase1 is required for the normal structure and behaviour of self-assembled spindles (Fig. 2), together with its location at the spindle midzone (Pineda-Santaella and Fernández-Álvarez, 2019), strongly suggest the existence in self-assembled spindles of a central zone composed of overlapping antiparallel microtubules. The location of Ase1 at the midzone can be explained by two plausible spindle microtubule configurations: (1) microtubule plus ends at the midzone and minus ends at the tips (SPB-mediated spindle polarity) or the opposite, (2) microtubule minus ends at the midzone and plus ends at the tips, similar to that of interphase microtubule arrays.

**Fig. 3.**
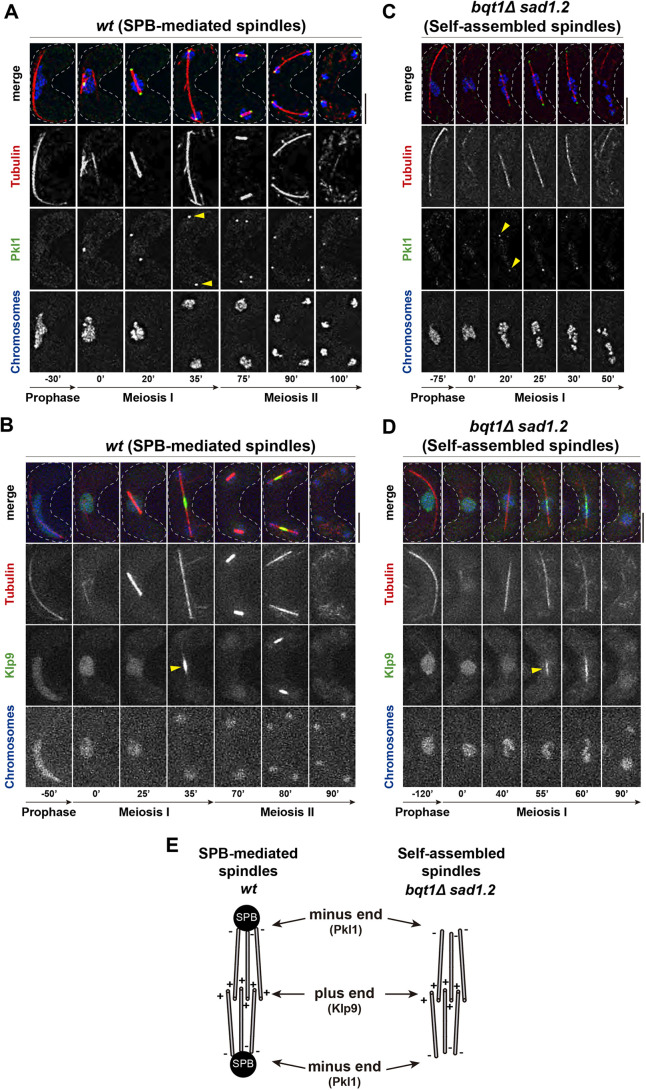
**SPB-dependent and self-assembled spindles share a similar polarity.** (A–D) Frames from films of meiocytes with endogenously GFP-tagged Pkl1 (in A and C) and Klp9 (in B and D); chromosomes and spindles tagged as in Fig. 1. Numbering indicates meiotic progression in minutes; *t*=0 is just before spindle formation. Scale bars: 5 µm. Yellow arrowheads indicate dots of Pkl1 and Klp9 patches. More than 15 cells from three independent experiments were analysed, showing in all cases the phenotypes represented. (E) Schematic of the proposed polarity of self-assembled spindles compared with that of the canonical SPB-mediated spindles.

To establish the polarity of self-assembled spindles, we analysed the localization of GFP-tagged spindle polarity markers Pkl1 (also known as Klp1)/kinesin-14 (Pkl1–GFP) and Klp9–GFP/kinesin-6 (Klp9–GFP), two motor kinesins that specifically track spindle microtubule minus ends and plus ends, respectively (Pidoux et al., 1996; Yukawa et al., 2015). As reported for SPB-mediated mitotic spindles, Pkl1–GFP localized to the spindle poles in MI (Fig. 3A, 20′ to 35′) and MII (Fig. 3A, 75′ to 90′), while Klp9–GFP localized to the spindle midzone in MI (Fig. 3B, 35′) and MII (Fig. 3B, 80′). Similarly, for self-assembled spindles, Pkl1–GFP localized to the spindle poles (Fig. 3C, 20′ to 30′; compare Fig. 3A, 35′ and C, 20′, yellow arrowheads) and Klp9–GFP localized to the spindle midzone (Fig. 3D, 55′ to 60′; compare Fig. 3B, 35′ and D, 55′, yellow arrowheads), indicating that the tips of self-assembled spindles are composed of microtubule minus ends and the midzone is composed of microtubule plus ends. Congruent with our previous results, the location of Pkl1 at the spindle poles in *bqt1*Δ *sad1.2* cells is SPB independent (Fig. S3). Hence, structural polarity of self-assembled spindles seems to obey the microtubule arrangement with minus ends at the poles and plus ends at the midzone, resembling that of SPB-mediated mitotic and meiotic spindles (Fig. 3E).

### F-actin networks are dispensable for self-assembled spindle formation and behaviour

To further characterize the formation and behaviour of self-assembled spindles, we explored the possible role of other cytoskeleton components. F-actin is essential for correct chromosome segregation in oocytes (Mogessie and Schuh, 2017). In fission yeast, F-actin networks are necessary for proper spindle orientation in mitosis (Gachet et al., 2001, 2006), and it has been observed that microtubule-independent nuclear movement requires actin cables (Ashraf et al., 2021). In meiosis, disruption of F-actin impairs, but does not completely abolish, chromosome segregation into four daughter nuclei (Petersen et al., 1998). We explored whether the F-actin network is important for the formation and behaviour of SPB-dependent and self-assembled spindles during fission yeast meiosis *in vivo*. For this purpose, we partially disrupted the F-actin network specifically during meiosis using the actin-depolymerizing drug latrunculin A (LatA) (see Materials and Methods) and then analysed spindle behaviour. We sought only partial disruption in order to not compromise the normal progression of meiosis and to avoid pleiotropic effects derived from complete F-actin elimination. To visualize the network, we fluorescently labelled F-actin using Lifeact (Riedl et al., 2008) and monitored it together with chromosomes and microtubules. In the absence of LatA, F-actin was observed as (1) cables (Fig. S4A, −100′, −75′ and 0′, red boxes and magnifications, yellow arrowheads), (2) numerous patches that fluctuated throughout the cell body during prophase and MI (Fig. S4A, −100′ to 35′), and (3) meiotic actin rings, which assembled around post-MI nuclei during MII (Fig. S4A, 85′ to 90′, see yellow arrowheads and cartoon) and eventually contracted (Fig. S4A, 95′) and disassembled, congruent with a previous description of F-actin in fixed meiocytes (Yan and Balasubramanian, 2012). In the absence of LatA, SPB-dependent spindles displayed normal assembly, elongation and disassembly behaviour, as well as symmetrical segregation of chromosomes into four masses of equal size (Fig. S4A, 0′ to 95′). On addition of 4 µM LatA, actin cables disappeared almost completely (95% reduction; compare Fig. S4A, −100′, −75′, 0′ with B and C). Also, the occurrence of normal meiotic actin rings was greatly reduced (from 100% to 14%); in the remaining cases, the meiotic actin rings were deficient (19%; Fig. S4B, 145′ to 180′, see yellow arrowheads and cartoon) or not formed at all (67%; Fig. S4C). These defects confirm that LatA was bioactive in our experimental conditions, achieving partial F-actin depolymerization throughout the whole meiosis.

**Fig. 4.**
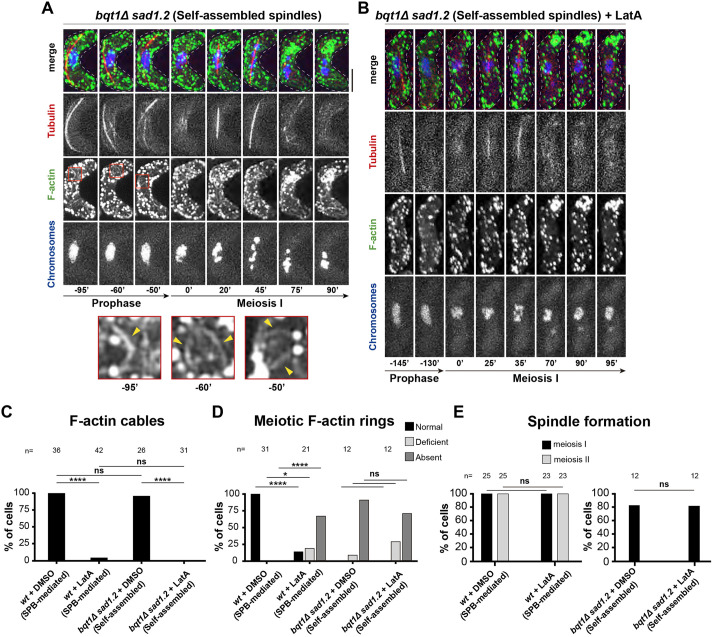
**Self-assembled spindle formation and behaviour are independent of the F-actin network in meiosis.** (A,B) Frames from films in meiosis. Numbers underneath represent time (in min) from MI onset. Scale bars: 5 µm. F-actin networks viewed via Lifeact–GFP, chromatin via histone H3 tagged at one of the two endogenous *hht1^+^* loci, and tubulin via ectopically expressed mCherry–Atb2 (also known as mCherry–Tub1). *bqt1*Δ *sad1.2* meiocytes in the presence (B) and absence (A) of latrunculin A (LatA) are shown. Red boxes: magnified sections of frames containing actin cables. Yellow arrowheads depict individual actin cables. (C–E) Comparison of the phenotypes shown by *wt* and *bqt1*Δ *sad1.2* meiocytes in the presence and absence of LatA, regarding F-actin cables (C), meiotic F-actin rings (D) and spindle formation (E). *n* is the total number of cells analysed from three independent experiments. Fisher's exact test: ****P<0.0001; *P<0.05; ns, *P*>0.05.

Next, we analysed the potential role of F-actin in self-assembled spindle formation and behaviour. In the absence of LatA, the behaviour of F-actin patches (Fig. 4A, −95′ to 0′) and cables (Fig. 4A, −95′ to −50′, red boxes and magnifications) was comparable to that seen for SPB-mediated spindles. However, no normal meiotic actin rings were observed; 9% of cells showed defective meiotic actin rings and 91% showed no meiotic actin rings, suggesting that loss of SPB insertion caused meiotic actin ring defects independently of LatA. Upon treatment with 4 µM LatA, actin cables and ring defects were comparable to those in the SPB-mediated setting (Fig. 4C,D). Notably, the formation and behaviour of self-assembled spindles were similar to those observed in the absence of LatA (Fig. 4B, 0′ to 35′; quantification in Fig. 4E), indicating that F-actin disruption did not have an impact on self-assembled spindle formation. Taken together, these findings show that the meiotic F-actin network does not play a key role in either the formation or the behaviour of self-assembled spindles.

### The γ-tubulin complex is absent in self-assembled spindle poles

**Fig. 5.**
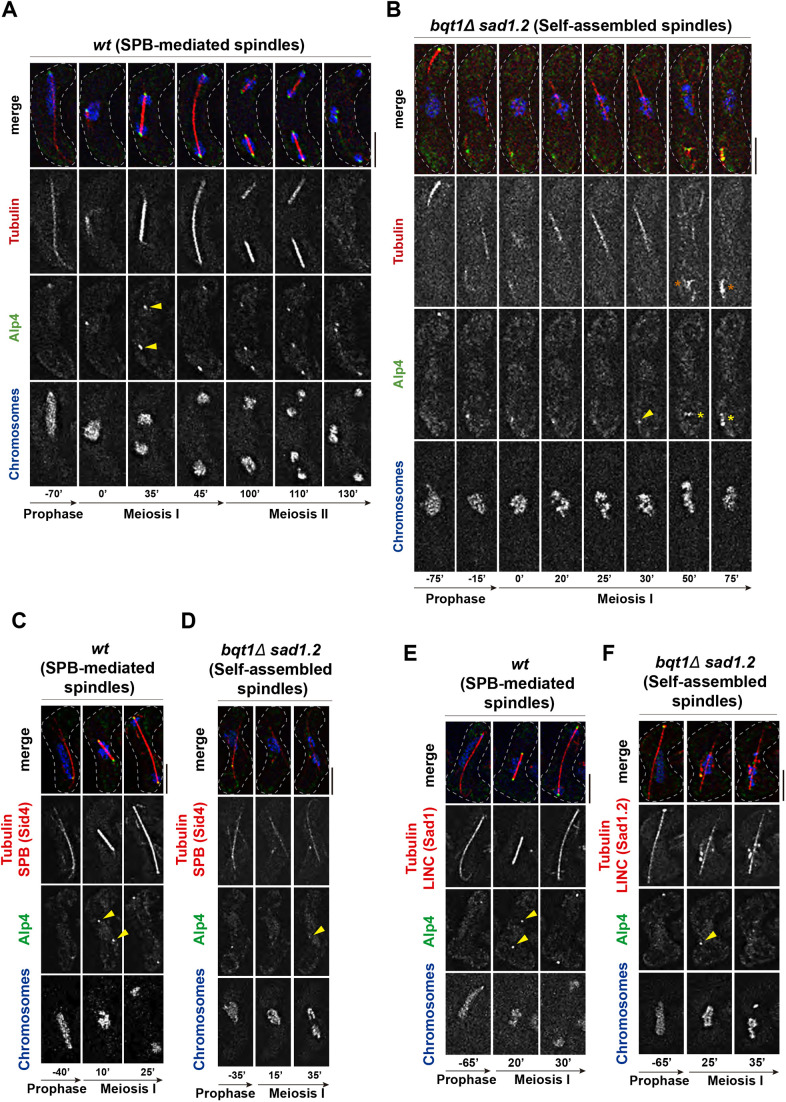
**Self-assembled spindle formation is independent of SPB-associated γ-tubulin complex.** (A–F) Frames from films of meiocytes carrying mCherry–Atb2 (ectopically expressed, tubulin), Hht1–CFP (at one of the two endogenous *hht1^+^* loci) and Alp4–GFP (endogenously expressed, gamma tubulin complex). Yellow arrowheads indicate dots of Alp4–GFP. Yellow asterisks indicate multiple Alp4 dots coming from an individual Alp4 dot. Orange asterisks indicate the appearance of microtubule polymerization in the vicinity of Alp4 dots. (A,B) 24 (*wt*) (A) and 29 (*bqt1*Δ *sad1.2*) (B) cells from three independent experiments were analysed, showing in all cases the phenotypes represented. (C–F) Sid4–mCherry (endogenously expressed) (C,D), Sad1–mRFP (endogenously expressed) (E) and Sad1.2–mCherry (endogenously expressed) (F) were used to visualize SPBs and LINC. Scale bars: 5 μm.

After completion of meiotic prophase, cytoplasmic oscillating astral microtubules dissolve just before the formation of SPB-mediated MI and MII spindles. Nucleation of spindle microtubules is carried out by a macromolecular protein complex called the γ-tubulin complex, which serves as a structural template for priming the *de novo* synthesis of microtubule filaments (Moritz et al., 2000). At the time of spindle nucleation, this complex is actively targeted to the nuclear side of inserted SPBs, where it nucleates microtubules that are then elongated via polymerization to project from the SPBs towards the nucleoplasm (Vardy, 2000; Bestul et al., 2017). In the case of self-assembled spindles, the SPBs are not inserted into the NE; thus, the possible role of the γ-tubulin complex is unclear. To investigate whether self-assembled spindle nucleation depends on this mechanism, we used a GFP-tagged version of Alp4 (Alp4–GFP), an essential component of the γ-tubulin complex (Vardy, 2000), as a proxy to monitor the localization of the complex relative to the self-assembled spindles. For SPB-mediated spindles, during meiotic prophase, one Alp4–GFP dot localized to the leading edge of the astral microtubule structure and followed its oscillating movement (Fig. 5A, −70′). After prophase, one dot of Alp4–GFP colocalized with the microtubule focus, from which the spindle emerges at the time of its formation (Fig. 5A, 0′), consistent with the description of γ-tubulin complex recruitment to the SPBs (Horio et al., 1991; Masuda et al., 2013). Later, concomitantly with MI spindle assembly and elongation, the original Alp4–GFP dot split into two dots, which perfectly colocalized with the spindle poles (Fig. 5A, 0′ to 45′). After MI spindle disassembly, the same behaviour was observed for MII SPB-mediated spindles (Fig. 5A, 100′ to 110′). Eventually, after MII spindle disassembly, each of the four resulting Alp4–GFP dots remained localized to each of the four resulting chromosome masses (Fig. 5A, 130′). For self-assembled spindles, Alp4–GFP similarly followed the leading edge of the oscillating astral microtubule structure. However, unlike in the SPB-mediated spindle background, the nucleus did not follow the oscillations (Fig. 5B, −75′). In contrast to the SPB-mediated setting, after prophase, while the self-assembled spindle formed within the chromosomal environment, no Alp4–GFP dots seemed to localize to the self-assembled spindle (Fig. 5B, 0′ to 30′; compare Fig. 5A, 35′ and B, 30′, yellow arrowheads). Furthermore, in a subset of cells, the original Alp4–GFP dot was observed to split into four dots (Fig. 5B, 50′ to 75′, yellow asterisks) and microtubules appeared in their vicinity (Fig. 5B, 50′ to 75′, orange asterisks), suggesting that these dots corresponded to the association of the γ-tubulin complex with the uninserted, dislodged SPBs. To confirm this, we evaluated the behaviour of *bqt1*Δ *sad1.2 alp4-GFP* cells harbouring the SPB markers Sid4–mCherry and Sad1.2–mCherry, effectively showing that Alp4–GFP molecules located far from the nucleus were associated with the SPBs (Fig. 5C,D) and the LINC complex (Fig. 5E,F). Thus, this lack of evident association between the self-assembled spindle poles and the Alp4–GFP dots, as seen in the SPB-mediated setting, suggests that the nucleation of self-assembled spindles may be independent of the conventional nucleation mechanism driven by the SPB-associated γ-tubulin complex.

### The TOG/XMAP215 microtubule polymerase Alp14 is a key factor for self-assembled spindle formation

Next, we investigated the possible role of other factors involved in microtubule nucleation and polymerization in controlling the formation of self-assembled spindles. In fission yeast, the TOG/XMAP215 family of microtubule polymerases is represented by two well-studied orthologues: Alp14 (Al-Bassam et al., 2012) and Dis1 (Nakaseko et al., 2001). Alp14 participates in the nucleation of interphase microtubule arrays (Flor-Parra et al., 2018a) and mitotic spindle formation, thereby contributing to SPB separation and correct bipolar spindle assembly (Yukawa et al., 2017). With the aim of exploring the behaviour of Alp14 during fission yeast meiosis and, in particular, in a self-assembled spindle scenario, we monitored a GFP-tagged version of Alp14 (Alp14–GFP) throughout meiosis. For SPB-mediated and self-assembled spindles, during prophase, Alp14–GFP localized to oscillating astral microtubules as dots (Fig. S5A, −45′ and B −25′, yellow arrowheads). At the onset of SPB-mediated spindle formation, Alp14–GFP colocalized with the tubulin focus, from which the spindle emerged (Fig. S5A, 0′, yellow arrowhead), suggesting a role for this polymerase in spindle microtubule nucleation. Once the spindle formed, Alp14–GFP showed several localization patterns: (1) first, as dots along the body of early short MI spindles (Fig. S5A, 15′), as has been reported for the mitotic spindle (Garcia, 2001; Al-Bassam et al., 2012); (2) second, to the poles (Fig. S5A, 30′, yellow arrowheads) and midzone (Fig. S5A, 30′, yellow asterisk) of late elongated MI spindles (Fig. S5A, 30′); and (3) to the whole body of MII spindles in a patched manner (Fig. S5A, 85′). Notably, Alp14–GFP also localized to self-assembled spindles (Fig. S5B, 20′, yellow arrowheads), similar to the behaviour observed in SPB-mediated spindles. These findings suggest that Alp14 might contribute to the formation and behaviour of self-assembled spindles.

To further characterize the nuclear signal of Alp14–GFP in the context of self-assembled spindle formation, we analysed Alp14–GFP nucleoplasmic fluorescence intensity throughout meiosis. Alp14–GFP accumulated in the nucleus at the end of prophase, as evidenced by an increasing signal colocalizing with chromosomes, prior to spindle formation in both settings (Fig. S5A, −20′ to 0′ and B, −25′ to 0′). Interestingly, the time interval between the initiation of Alp14–GFP nuclear accumulation and spindle formation onset was longer for self-assembled spindles than for SPB-mediated spindles (10±2 min difference; Fig. S5C). To check whether this difference was a consequence of abnormal Alp14 dynamics, we estimated the amount of nuclear Alp14 in this time window by measuring Alp14–GFP total intensity within the chromosomal environment (see Materials and Methods). This quantification showed no significant difference between the total levels of nuclear Alp14–GFP in both settings (Fig. S5D), suggesting that pre-meiotic Alp14 nuclear accumulation remained normal, and so suggesting a delay in self-assembled spindle formation, which is consistent with previously observed defective spindle biophysics (Pineda-Santaella and Fernández-Álvarez, 2019).

**Fig. 6.**
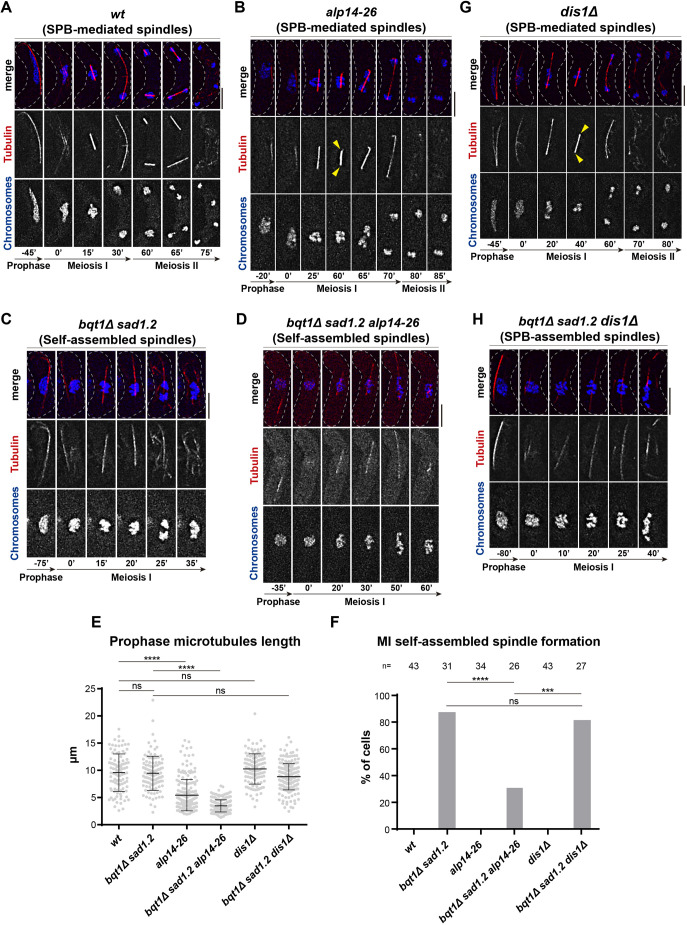
**Loss of Alp14 leads to a smaller percentage of self-assembled spindles.** (A–D,G,H) Frames from films of meiocytes carrying chromosomes and spindles tagged as in Fig. 1. Numbering indicates meiotic progression in minutes; *t*=0 is just before spindle formation. Scale bars: 5 µm. All the experiments were performed at 28°C. Yellow arrowheads depict spindle behaviour defects. (E) Quantification of prophase microtubules length from the genotypes shown in A–D, G and H. Mann–Whitney test: ****P<0.0001; ns, *P*>0.05. (F) Quantification of self-assembled spindle formation frequency from the genotypes shown in A–D, G and H. *n* is the total number of cells analysed from three independent experiments. Fisher's exact test: ****P<0.0001; ***P<0.001; ns, *P*>0.05.

To ascertain whether Alp14 plays a crucial role or, alternatively, whether self-assembled spindle formation does not require Alp14, we tried to generate the triple-deletion mutant *bqt1*Δ *sad1.2 alp14*Δ. However, we found that loss of *alp14* led to severe growth defects in *bqt1*Δ *sad1.2* cells, as checked by tetrad analysis (Fig. S5E), and we were unable to film meiosis in this triple mutant. To circumvent this problem, we used the thermosensitive allele *alp14-26* (Yukawa et al., 2019). Meiosis analysis cannot be performed at temperatures above 32°C, but analysis of *alp14-26* meiosis at a semi-permissive temperature of 28°C showed defects in prophase microtubules length (compare Fig. 6A, −45′ with D, −35′ and Fig. S5F, −25′; see quantification in Fig. 6E) and SPB-mediated spindle formation (compare Fig. 6A with B and Fig. S5F, yellow arrowheads). This partial loss of function indicates that *alp14-26* is more thermosensitive in meiosis than in mitosis. Remarkably, we found that, in the case of *bqt1*Δ *sad1.2 alp14-26* meiocytes, despite clear dysfunction of *alp14-26*, self-assembled spindles were still able to form and behaved normally (Fig. 6C,D). However, they formed in a smaller percentage of meiocytes (from ∼80% to ∼30%, Fig. 6F), indicating that the contribution of Alp14 to self-assembled spindle formation and behaviour is substantial.

To further define the role of the TOG/XMAP215 family in self-assembled spindle formation, we investigated the role of Dis1. In contrast to deletion of Alp14, loss of Dis1 in *bqt1*Δ *sad1.2* cells led to slight defects in vegetative growth, which allowed us to analyse self-assembled spindles in *bqt1*Δ *sad1.2 dis1*Δ triple mutant meiosis (Fig. S5G). Although deletion of *dis1* in the SPB-dependent spindle setting led to severe defects in spindle formation and function (Fig. 6G and Fig. S5H, yellow arrowheads), analysis of *bqt1*Δ *sad1.2 dis1*Δ meiocytes showed that the percentage of self-assembled spindles with normal formation and function was similar to that in the *bqt1*Δ *sad1.2* setting (Fig. 6F,H). Taken together, these data indicate that the TOG/XMAP215 microtubule polymerase Alp14 is a key factor for self-assembled spindles, and that its contribution to spindle formation and behaviour is higher for self-assembled spindles than for canonical SPB-dependent spindles.

### The robustness of self-assembled spindles improves with the loss of kinesin-8

An important characteristic of self-assembled spindles in *bqt1*Δ *sad1.2* meiocytes is their low robustness, which is likely the reason for the observed high rate of chromosome segregation defects. The weakness of the self-assembled spindle structure is more common in the second meiotic divisions, as the majority of meiocytes do not form MII spindles. These defects make it difficult to study the molecular mechanism behind SPB-independent spindle formation, especially in meiosis II. To gain insight into the molecular basis of acentrosomal meiosis, we tried to improve the robustness of self-assembled spindles. We explored different strategies, the most successful of which was the deletion of *klp6*. Klp5 and Klp6, the representatives of the kinesin-8 family in fission yeast, are motor proteins that form a heterodimer, Klp5–Klp6, which is involved in regulating microtubule dynamics in interphase and mitosis through diverse activities (Unsworth et al., 2008; Gergely et al., 2016; West and McIntosh, 2008) such as microtubule destabilization. Indeed, deletion of either protein leads to depolymerization-resistant interphase microtubules (Garcia et al., 2002) and promotes spindle microtubule polymerization (Pinder et al., 2019). To assess the potentially improved robustness of the spindle, we quantified the effect of Klp6 elimination according to three parameters: (1) spindle formation frequency, (2) maximum spindle length as a readout of structural strength and (3) chromosome segregation efficiency.

**Fig. 7.**
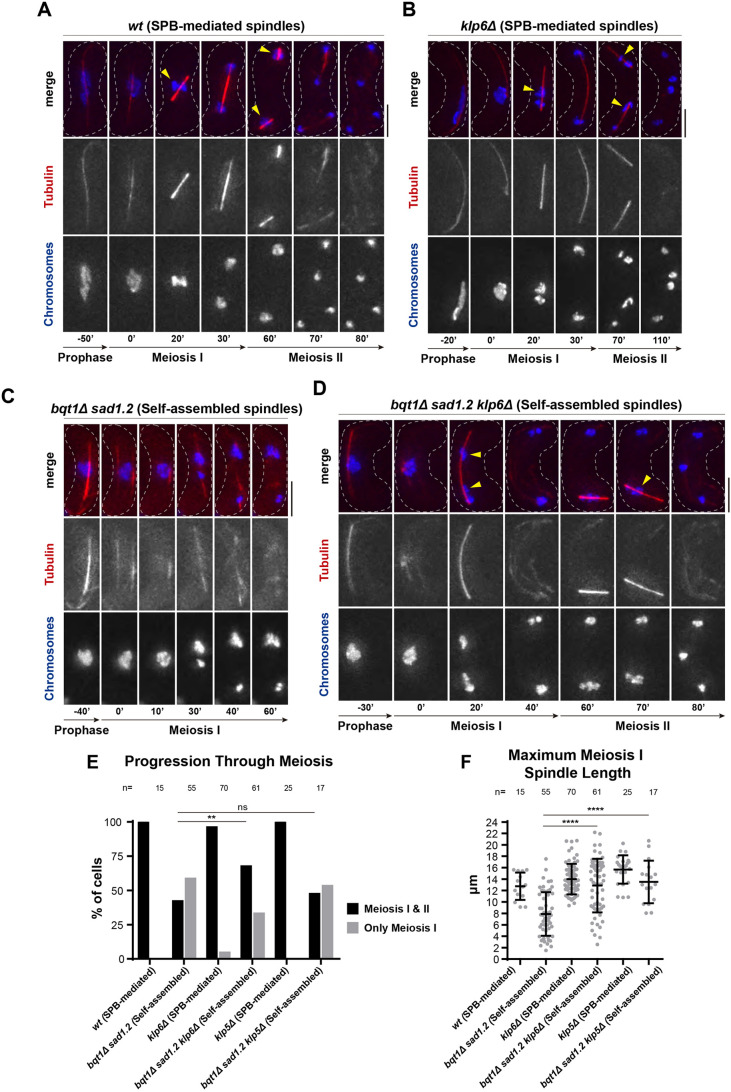
**Elimination of kinesin-8 Klp6 improves the formation, structure and chromosome segregation of self-assembled spindles.** (A–D) Frames from films of meiocytes carrying chromosomes and spindles tagged as in Fig. 1. Numbering indicates meiotic progression in minutes; *t*=0 is just before spindle formation. Scale bars: 5 µm. Yellow arrowheads indicate the positioning of chromosome mass(es) with respect to the spindle structure before being segregated in MI and MII. (E) Quantification of cells showing SPB-mediated spindles and self-assembled spindles only in MI and in MI+MII. (F) Quantification of maximum MI spindle length. Bars represent mean and s.d. *n* is the total number of cells scored from more than three independent experiments. Fisher's exact test ****P<0.0001; **P<0.01; ns, *P*>0.05.

The absence of Klp6 did not affect MI and MII SPB-mediated spindle formation (compare Fig. 7A and B), although it did produce previously reported chromosome segregation defects, including chromosomes remaining asymmetrically positioned along the spindle axis before segregation (Fig. 7B, 20′, yellow arrowhead, and 70′, top yellow arrowhead) as well as lagging chromosomes (Fig. 7B, 70′, bottom yellow arrowhead) (Pinder et al., 2019; Syrovatkina et al., 2013). Remarkably, elimination of Klp6 rendered MI and MII self-assembled spindles thicker and brighter than those formed in the *klp6^+^* setting (compare Fig. 7C, 30′ and D, 20′; Fig. S6), suggesting an increase in the robustness of the spindle, although chromosome segregation defects persisted (Fig. 7D, 20′ and 70′, yellow arrowheads). To further substantiate this observation, we quantified spindle formation frequency and showed that *klp6* deletion increased the percentage of cells displaying MII self-assembled spindles from 42% to 67% (with respect to the total number of cells undergoing MI; *n*=55 and *n*=61, respectively), thus promoting spindle formation and progression to MII (Fig. 7E). Conversely, there was no significant difference between *klp6^+^* and *klp6*Δ MII SPB-dependent spindle formation rate (both ∼100%), which means that the absence of Klp6 does not significantly affect SPB-dependent meiosis progression (Fig. 7E). Moreover, we studied the effect of Klp6 on spindle structure by measuring the maximum length of MI spindles. Remarkably, the maximum length of self-assembled spindles increased upon deletion of *klp6* (from 7.9±3.8 µm to 12.9±4.7 µm; Fig. 7E). This indicates that elimination of Klp6 provokes the strengthening of self-assembled spindle structure.

Next, we analysed the rate of chromosome segregation defects in self-assembled spindles in cells with and without Klp6. In particular, we quantified the number of chromosome masses after MI segregation. Most *klp6*Δ SPB-dependent spindles (84%) segregated parental chromosomes into two masses, i.e. normal segregation, although a minority exhibited three to six masses, consistent with chromosome segregation defects intrinsic to the loss of Klp6 (Fig. S7A, *n*=69) (Pinder et al., 2019; Syrovatkina et al., 2013; Garcia et al., 2002; West et al., 2001). Self-assembled spindles presented severe segregation defects: nearly a third (32%) of the spindles did not segregate chromosomes, leaving the parental nucleus as a single mass, while another third (36%) segregated chromosomes into up to three to six masses, and the remaining spindles (32%) segregated chromosomes into two masses (Fig. S7A, *n*=57). Strikingly, after *klp6* deletion, the rate of chromosome mis-segregation decreased, from 32% to 0% for single masses and from 19% to 6% for four to six masses. Consequently, the percentage of normal segregation rose from 32% to 61%, and the percentage of mild mis-segregation, i.e. three masses, rose from 18% to 32% (Fig. S7A, *n*=62). Hence, elimination of Klp6 increased the fidelity of chromosome segregation for self-assembled spindles, reducing the incidence of aneuploidy and illustrating that spindle improvement occurred not only at the level of formation and structure, but also at the functional level.

Although individual deletions of Klp5 and Klp6 equally lead to the loss of Klp5–Klp6 heterodimer activity, loss of function of each component exerts distinct effects on spindle microtubule dynamics, in part owing to differential contributions of unique functional domains within each partner (Gergely et al., 2016; Unsworth et al., 2008; West and McIntosh, 2008). Thus, to account for the possibility that the effect of Klp5 deletion on self-assembled spindles is different from that of Klp6, we repeated the analysis on *klp5^+^* and *klp5*Δ SPB-dependent and self-assembled spindles in meiosis. Similar to deletion of Klp6, loss of Klp5 increased the maximum length of self-assembled spindles (Fig. 7F); however, in contrast to Klp6 deletion, Klp5 deletion also increased the maximum length of SPB-mediated spindles (Fig. 7F), as described previously (Flor-Parra et al., 2018b). Nonetheless, Klp5 deletion did not cause a significant improvement either in progression to MII in *bqt1*Δ *sad1.2* meiocytes (Fig. 7E) or in chromosome segregation fidelity for self-assembled spindles (Fig. S7A). Thus, elimination of Klp5 reinforced self-assembled spindle structure but, unlike Klp6 deletion, it did not have a positive impact on self-assembled spindle formation or function.

Taken together, these data show that loss of kinesin-8, specifically, Klp6, but not Klp5, is sufficient to improve self-assembled spindle structure, promote its formation and increase its chromosome segregation efficiency. Deletion of *klp6* thus represents a genetic optimization for this fission yeast system, in the sense that it facilitates the study of MII, enables finer analysis of self-assembled spindle structure and dynamics, and harbours more efficient chromosome segregation, better resembling that of mammalian acentrosomal meiosis.

## DISCUSSION

**Fig. 8.**
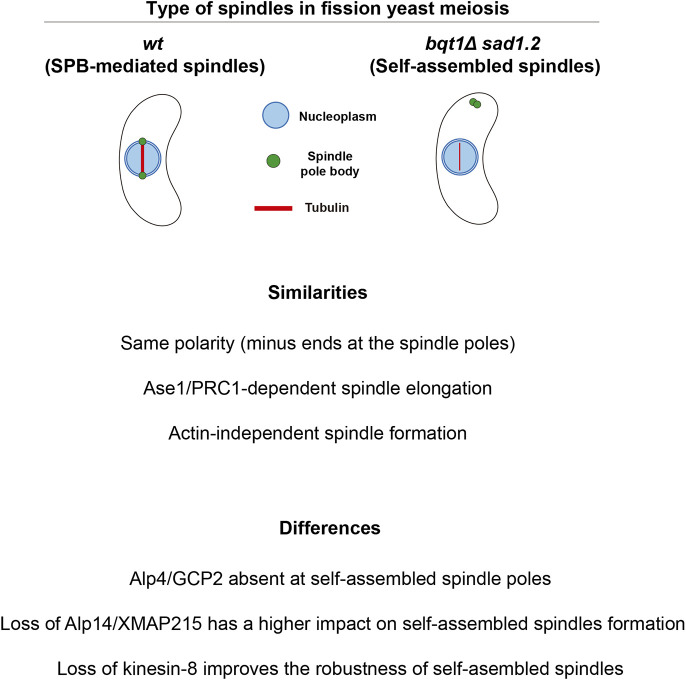
**Differences and similarities between SPB-mediated and self-assembled spindles in fission yeast meiosis.** Schematic and summary of the main features of spindle self-assembly in *bqt1*Δ *sad1.2* meiocytes.

In this work, we carried out a molecular characterization of an unexpected type of self-assembled spindle in fission yeast meiosis, which allowed us to establish the main similarities and differences between SPB-dependent and SPB-independent spindles (Fig. 8). Their similarities include structural dependence on microtubule crosslinker Ase1/PRC1, the polarity of microtubule arrangement, and the recruitment of the TOG/XMAP215 microtubule polymerase Alp14. In terms of their differences, self-assembled spindle formation seems to be independent of conventional spindle nucleation involving the γ-tubulin complex, and a member of the TOG/XMAP215 family, Alp14, seems to have a major role in self-assembled spindle behaviour. Moreover, deleting the kinesin-8 Klp6 increased the robustness of self-assembled spindles by improving their formation, structure and chromosome segregation fidelity, but it had a neutral or negative impact on SPB-dependent spindles. In addition to providing valuable information in the present study, our improvement of self-assembled spindles will be useful for future studies aiming to understand the molecular basis of acentrosomal meiosis using fission yeast as a model organism.

### SPB-independent self-assembled microtubules behave as a proper spindle

A major question in our work is whether the self-assembly of nuclear microtubules in the absence of SPB insertion into the NE follows the characteristics of SPB-mediated spindle formation. In this work, we confirm the presence of spindle-like properties; for instance, we show that self-assembled spindles require the canonical microtubule crosslinker Ase1/PRC1 to maintain their structural stability. Ase1 regulates the dynamics of both interphase and mitotic/meiotic microtubule arrays; deletion of *ase1* causes misorientation and misconfiguration of interphase arrays (Loïodice et al., 2005), whereas its deletion in mitosis leads to breakage of the spindle body at late stages of spindle elongation (Yamashita et al., 2005), consistent with our observations for both meiotic SPB-mediated and self-assembled spindles. Congruent with our data, recent observations have established that Ase1 and microtubule motors together are sufficient to form spindle-like structures independently of the presence of microtubule-organizing centres (Hannabuss et al., 2019; Blackwell et al., 2017).

Despite the wide range of configurations permitted by microtubule versatility, the structure and polarity adopted by self-assembled microtubules resemble those of SPB-dependent spindles. Microtubule minus ends face outwards at the spindle poles and plus ends project inwards at the midzone towards chromosomes, as shown by the localization pattern of the kinesins Pkl1 and Klp9, respectively. We hypothesize that self-assembled spindle polarity is the same as that of centrosomal spindles, owing to a set of microtubule-associated factors that force microtubules to adopt the canonical spindle-type configuration instead of an alternative configuration, such as that of interphase arrays. Microtubule configuration within the spindle has fundamental implications for the mechanism of chromosome segregation. We speculate that the mechanism is likely to involve chromosome capture by microtubule plus ends and then pulling to opposite poles driven by microtubule shrinkage, as for SPB-mediated spindles (Tanaka et al., 2005). In addition, the poleward movement of chromosomes that we observed could be complemented by supporting mechanisms such as pushing forces powered by microtubule polymerization (Ault et al., 1991), as reported for *C. elegans* meiosis (Laband et al., 2017), as well as microtubule sliding (Vukušić et al., 2017).

### Self-assembled spindle formation is independent of F-actin

In mammalian oocytes, F-actin cooperates with spindle microtubules to assist in the polarization and maintenance of spindle structure (Roeles and Tsiavaliaris, 2019), drives the necessary asymmetrical spindle positioning within the oocyte (Mogessie et al., 2018) and helps to ensure correct chromosome segregation (Mogessie and Schuh, 2017). Given the importance of F-actin for mammalian oocytes, we explored its relevance in acentrosomal meiosis in fission yeast. Disruption of the F-actin network in fission yeast meiosis revealed that it is not required for self-assembled spindle formation or behaviour.

Analysis of the F-actin network during meiosis showed that the *bqt1*Δ *sad1.2* setting has an intrinsic defect in meiotic actin ring formation. In *S. pombe* meiosis, the SPBs localize to opposite poles of each MII nuclei and serve as a reference point at which the forespore membrane starts to form (Hirata and Shimoda, 1994; Hagan and Yanagida, 1995; Tanaka and Hirata, 1982). Meiotic actin rings then assemble and contract in a coordinated manner with MII nuclear divisions, guiding the extension and wrapping of the membrane around each nucleus (Yan and Balasubramanian, 2012; Itadani et al., 2006; Ohtaka et al., 2007). Given that the SPBs seem to trigger forespore membrane formation and that the SPBs are completely detached from the NE in *bqt1*Δ *sad1.2* cells, meiotic actin rings may be unable to assemble properly around nuclei.

### How are self-assembled spindles nucleated in fission yeast meiosis?

Our observations suggest that conventional spindle nucleation by the SPB-associated γ-tubulin complex may not be involved in self-assembled spindle formation; we did not observe clear dot-like accumulation and association of one essential member, Alp4, at the poles or elsewhere on the self-assembled spindle structure, as is observed for SPB-mediated spindles. However, we cannot rule out the involvement of the γ-tubulin complex in self-assembled spindle formation via other pathways.

An alternative explanation for the absence of an Alp4 signal at the poles of self-assembled spindles might be linked to the fact that self-assembled spindles have a thinner structure than SPB-dependent spindles, thereby indicating the presence of fewer microtubules. We hypothesize that self-assembled spindles would then need a smaller amount of nucleation factors, including the γ-tubulin complex, than the canonical spindle requires. Hence, these factors would not be easily detectable by the live fluorescence microscopy methods used in this study. Nonetheless, the true absence of Alp4, and the γ-tubulin complex in general, near self-assembled spindles could also be possible. In fact, in mitotic cells in *C. elegans* embryos, about half the kinetochore microtubules, which contribute to building up the mitotic spindle, do not depend on the γ-tubulin complex to be nucleated (O'Toole et al., 2003). Similarly, microtubules forming the self-assembled spindle in fission yeast could be nucleated independently of the γ-tubulin complex via other microtubule nucleators, which would explain the absence of Alp4 around this type of spindle. This possibility is supported by the discovery that some microtubule-associated proteins can promote microtubule nucleation independently of the γ-tubulin complex (Wieczorek et al., 2015).

In addition to nucleation of microtubules, another essential feature for spindle assembly is microtubule polymerization – a process that aids SPB separation and spindle elongation (Yukawa et al., 2017) – by polymerases such as TOG/XMAP215 family members Alp14 and Dis1. The fact that Alp14 accumulates in the nucleus in the self-assembly context in a manner somewhat similar to that in SPB-dependent spindles reveals that nucleocytoplasmic traffic of Alp14 is still functional in the absence of SPB insertion. Another notable feature of Alp14 behaviour is its rather dispersed distribution inside the nucleus at the time of self-assembled spindle formation compared to that in the SPB-mediated spindle setting. We propose that because self-assembled spindles might be composed of fewer microtubules, fewer Alp14 molecules would be loaded into the spindle body and the remaining Alp14 molecules would remain free at the nucleoplasm, unable to load into the saturated microtubule lattice. Regarding Alp14 nuclear accumulation, one possibility is that its timing in the self-assembly setting is comparable to that in the wild type, meaning that self-assembled spindle formation is delayed with respect to SPB-mediated spindle formation. We predict that spindle self-assembly is less efficient in the absence of a reference point and organization from the SPBs and/or it requires more spindle assembly factors, displaying an inherent defectiveness that translates into more time needed to form. Alternatively, Alp14 accumulation in the self-assembly setting could be temporally different from that of the wild type, in which case further analysis would be required using temporal references that are different from those of spindle formation or SPB duplication (which would not be applicable due to SPB dislodgement).

Unfortunately, because *bqt1*Δ *sad1.2 alp14*Δ cells show severe growth defects, it is not trivial to explore the consequences of Alp14 absence in self-assembled spindles during fission yeast meiosis. We think that this growth impairment is a consequence of combined *alp14* and *sad1.2* misfunctions. Their genetic interaction is confirmed in this study and is consistent with that described between *alp14* and *csi1*, a partner of Sad1, which is necessary to stabilize centromere/telomere–Sad1 interactions and correct spindle formation and chromosome segregation (Hou et al., 2012). To overcome the defects of the triple mutation, we used the thermosensitive allele *alp14-26* at a semi-permissive temperature, revealing that the contribution of Alp14 to self-assembled spindle formation is critical. Unlike Alp14, its paralogue Dis1 appears to be disposable for self-assembled spindle formation and function, because its deletion has little, if any, effect on these processes. This sharp difference between Alp14 and Dis1 in their contribution to self-assembled spindles may reflect their different functions and the mechanisms they use to regulate microtubule dynamics (Garcia, 2001; Yukawa et al., 2019).

### Why does loss of kinesin-8 improve self-assembled spindles?

Elimination of the kinesin-8 Klp6, but not its partner Klp5, increases the stability of self-assembled spindles and consequently increases their formation rate, structural strength and efficiency of chromosome segregation in *bqt1*Δ *sad1.2* meiocytes. One of the roles of the kinesin-8 heterodimer Klp5–Klp6 is to destabilize microtubules by (1) promoting microtubule catastrophe (Erent et al., 2012; Unsworth et al., 2008), and (2) hampering polymerization and enhancing depolymerization (Garcia et al., 2002). A possible mechanism for increasing the thickness of self-assembled spindles is enhanced polymerization and elongation of free tubulin and/or microtubule fragments incorporated into the lattice of already formed microtubule filaments (Schaedel et al., 2019), thereby thickening the spindle body (Fig. S8A,B). The longer length of *klp6*Δ self-assembled spindles could be explained by microtubule overgrowth derived from enhanced polymerization, as well as by microtubule hyperstabilization, which would confer the spindle with resistance to the microtubule-destabilizing mechanisms responsible for spindle disassembly, helping it to reach a longer length (Fig. S8A,B). Enhanced polymerization would also promote self-assembled spindle formation, explaining the increase in the occurrence of self-assembled spindles in MII (Fig. S8B,C). Loss of Klp6 leads to chromosome segregation defects in an SPB-mediated setting (Fig. S7A), as previously described (Pinder et al., 2019; Syrovatkina et al., 2013; Garcia et al., 2002; West et al., 2002). However, in our system, elimination of Klp6 significantly improved chromosome segregation carried out by self-assembled spindles, probably as a consequence of reinforced spindle structure as well as stabilization of kinetochore-microtubule interaction, which might facilitate kinetochore capture (Fig. S8B). By contrast, elimination of Klp5 had a positive impact on self-assembled spindle structure, but this improvement did not extend to more important functions, like consistent spindle formation up to MII and reliable chromosome segregation, which is the fundamental purpose of the spindle. Therefore, we speculate that it may be more beneficial to the performance of self-assembled spindles to conserve the activity of Klp5 rather than that of Klp6. This difference between the effects of Klp5 and Klp6 deletion is consistent with other described discrepancies between *klp5*Δ and *klp6*Δ settings relative to the regulation of interphase microtubules (Unsworth et al., 2008) and also, of note, mitotic spindle microtubules (Gergely et al., 2016).

Hence, our results demonstrate a successful strategy for improving acentrosomal spindle structure and function in fission yeast meiosis (Fig. S8C). This strategy not only benefits self-assembled spindles formation from a practical, experimental perspective, but also brings it closer to mammalian acentrosomal meiosis, making it a more reliable platform for studying the molecular basis of this process.

## MATERIALS AND METHODS

### Strains and media

Strains used in this work are listed in Table S1. Media were as described in Moreno et al. (1991). Strains were thawed in a solid yeast extract supplemented (YES) rich medium plate and incubated for 24 h at 32°C or 48 h at 25°C and patched in a new fresh plate and incubated for 24 h at 32°C or 25°C. For meiosis induction, strains were repatched in solid sporulation agar (SPA) medium and incubated for 6–6.5 h at 28°C, the optimal temperature for mating and meiosis. After induction, meiotic cells were immobilized with lectin (0.2 µg/ml, Sigma-Aldrich, L1395) at the bottom of a µ-Dish glass-bottom 35 mm uncoated dish (81151, ibidi Gmbh), washed and covered in a total of 3 ml EMM2 minimal medium without nitrogen, with or without drug, to ensure continuation of meiosis.

### Live analysis

For acquisition of live fluorescence microscopy images, two microscopy systems were used: a spinning disk confocal microscope (Photometrics Evolve camera; Olympus 100× 1.4 NA oil immersion objective; Roper Scientific-Photometrics) and a Delta Vision (CoolSnap HQ camera; Olympus 100× 1.4 NA oil immersion objective, environmental temperature and CO_2_ precision control; Inverted Microscope Olympus IX71). For spinning disk confocal microscopy, images were taken using 14 *z*-sections separated by 0.5 µm every 5 min over 5–6 h, with the following channels, exposure times and laser intensities: mCherry (561 nm), 150 ms, 50%; GFP (491 nm), 100 ms, 25%; CFP (405 nm), 100 ms, 20%; brightfield (visible), 50 ms. For Delta Vision, images were taken using 15–20 *z*-sections separated by 0.4 µm every 5 or 10 min over 5–6 h, with the following channels, exposure times and radiation intensities: YFP (492 nm), 150 ms, 50%; CFP (436 nm), 100 ms, 32%; TRITC (555 nm), 500 ms, 100%; brightfield (visible), 200 ms, 10%. Maximum *z*-projections of acquired images were obtained and stacked with ImageJ software (version 1.52p) (http://rsbweb.nih.gov/ij/). Acquired images were deconvolved with the PSFs and scripts available at https://github.com/danilexn/deCU, based on the Richardson–Lucy algorithm implementation by Shao and Milkie, at the Betzig laboratory (https://github.com/scopetools/cudaDecon). Further image processing was performed using Adobe Photoshop CC 2018 and Adobe Illustrator CC 2017. Only meiotic cells that progressed normally into and along meiosis were submitted to analysis, discarding cells with (pre-)meiotic defects, such as non-fusion of parental nuclei (karyogamy) or viability defects, such as cell death during image acquisition.

### LatA treatment

To treat meiotic cells with LatA (Sigma-Aldrich, L5163), 12 µl of 1 mM LatA was mixed with 490 µl EMM2 minimal medium, and the resulting 500 µl was mixed with the remaining 2.5 ml EMM2 minimal medium used to cover cells during image acquisition. This acquired a final concentration of LatA in the total 3 ml medium of 4 µM. LatA was added to meiotic cells after meiosis induction, just at the start of the filming, and cells were incubated with the drug during the whole time of filming.

### Quantification and statistical analysis

Quantification of nuclear fluorescence signal of Alp14–GFP was performed by measuring in a maximum *z*-projection signal intensity within the nuclear environment, delimited by the perimeter described by the signal of the parental nucleus chromosomal mass before spindle formation. For prophase microtubules length, measurements of individual microtubules were taken during meiotic prophase using ‘Segmented Line’ tool in ImageJ.

Statistical tests were performed with GraphPad Prism 6. To test for difference between the means of two distributions, if both followed a normal distribution, a parametric unpaired Student's *t*-test (with or without Welch's correction, as indicated) was performed; otherwise, a non-parametric Mann–Whitney test was performed. To test for difference between two proportions, a Fisher's exact test was performed.

## Supplementary Material

Supplementary information

Reviewer comments
