# Supplementary material for: Loss of kinesin-8 improves the robustness of the self-assembled spindle in Schizosaccharomyces pombe
Source: J Cell Sci. 2021 Aug 23;134(16):jcs253799. doi: 10.1242/jcs.253799 (PMC8435293; doi:10.1242/jcs.253799)
Supplement: Supplementary information [file joces-134-253799-s1.pdf]

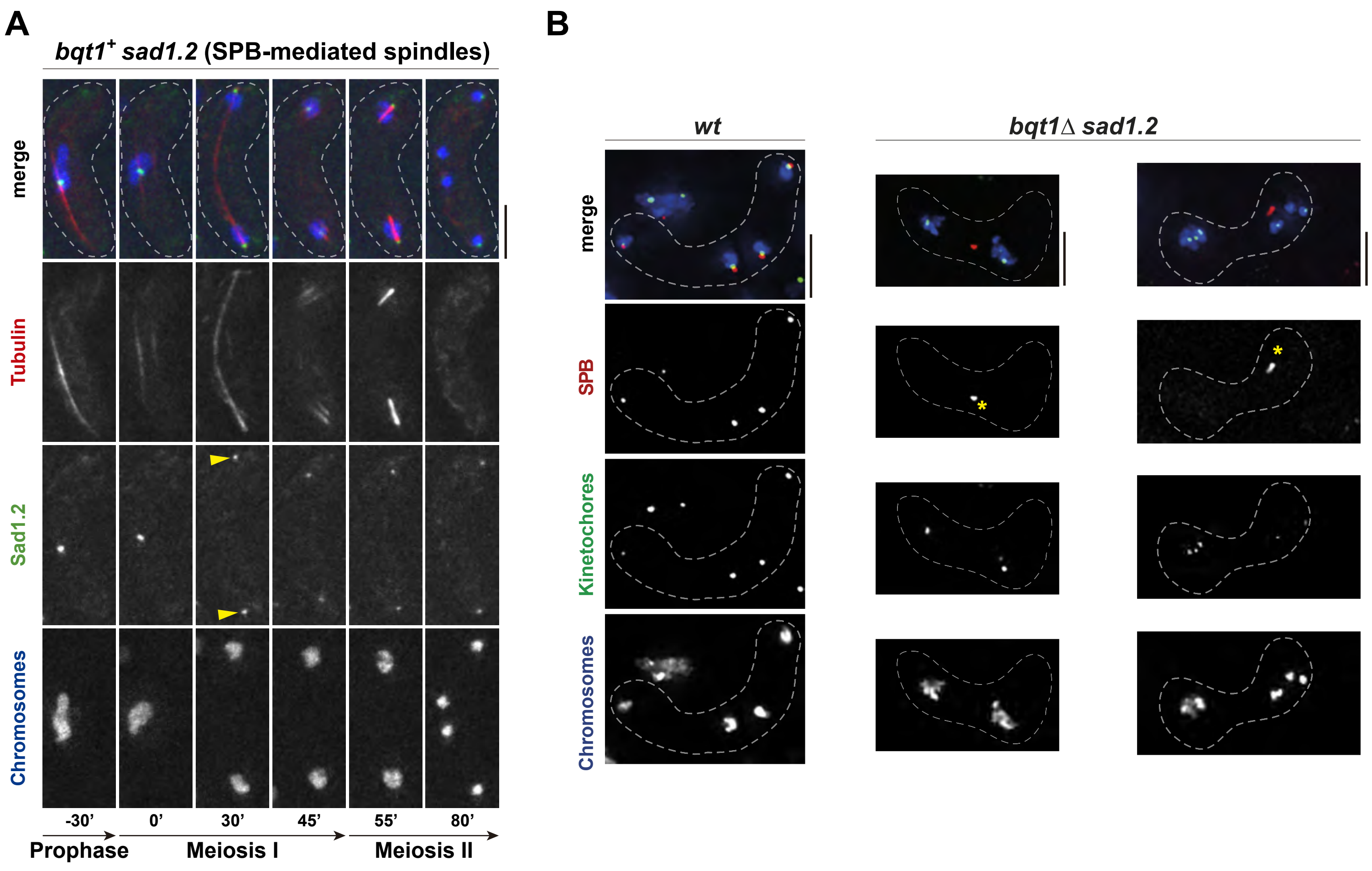

Fig. S1. (Complement to Figure 1). Chromosomes segregated in bqt1Δ sad1.2 meiocytes harbour kinetochore signal. (A) Frames from films of meiocytes carrying Hht1-CFP (at one of the two endogenous *hht1+* loci), ectopically expressed mCherry-atb2 (tubulin) and endogenously tagged Sad1.2-GFP. Numbering indicates meiotic progression in minutes; t = 0 is just before spindle formation. Sad1.2 localises at SPB-mediated spindle poles in 100% of *bqt1+ sad1.2* meiocytes (20 cells were analysed from more than two independent experiments). Yellow arrowheads indicate the location of Sad1.2-GFP during SPB-mediated spindle formation. (B) In *bqt1Δ sad1.2* cells, without SPB separation, segregated chromosomes harbour kinetochores. Snapshots of wt and bqt1Δ sad1.2 meiocytes after meiosis: kinetochore (Mis6-GFP), chromatin (Hht1-CFP), and the SPB (Sid4-mCherry) are visualized. Unseparated SPBs are indicated by the yellow asterisks. Bars represent 5 µm.

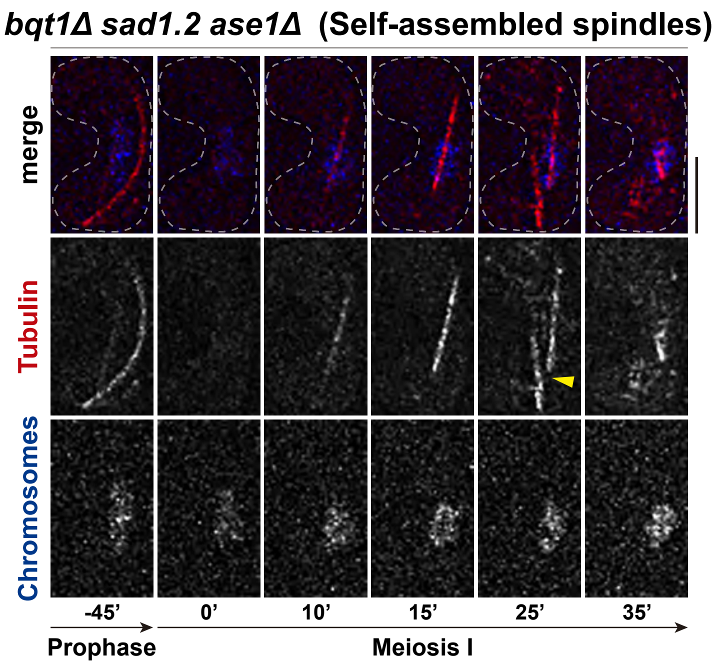

Fig. S2. (Complement to Figure 2). Ase1/PRC1 is an essential component of *bqt1Δ sad1.2* spindles. Frames from films of meiocytes carrying chromosomes and spindles tagged as in Figure 2. Numbering indicates meiotic progression in minutes; t = 0 is just before spindle formation. Bars represent 5 µm. Yellow arrowhead indicates discrete breakage of the spindle structure (25').

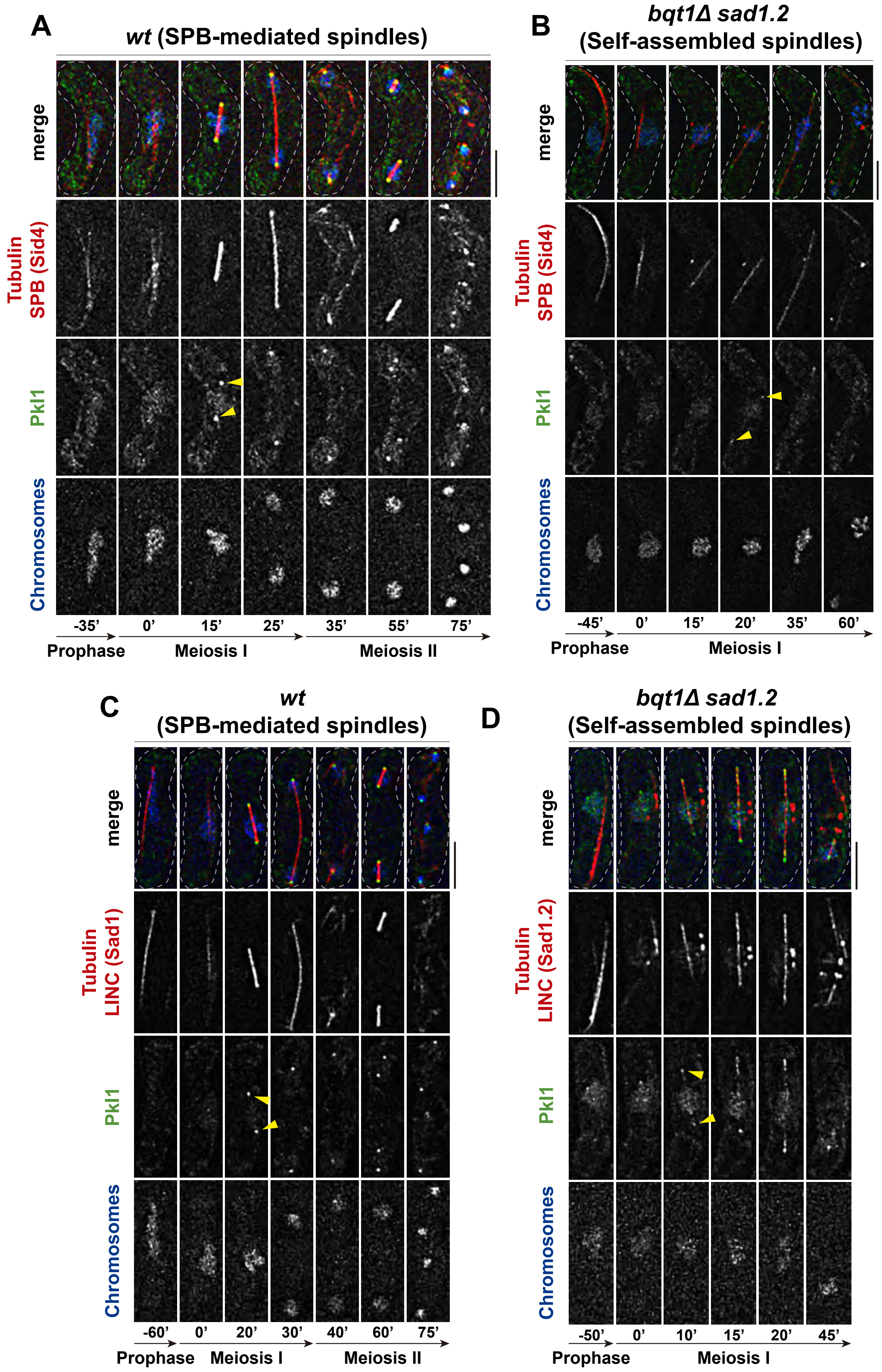

Fig. S3. (Complement to Figure 3). The location of Pkl1 at the spindle poles in *bqt1*Δ *sad1.2* cells is SPB-independent. (A-B) Frames from films of meiocytes with endogenously mCherry-tagged Sid4 and GFP-tagged Pkl1, chromosomes and tubulin are visualized as in Figure 3. (C-D) To visualize the LINC complex, Sad1 and Sad1.2 were endogenously tagged with RFP and mCherry, respectively. Sid4 or Sad1.2 and Pkl1 does not colocalise at self-assembled spindle poles in 100% of bqt1Δ sad1.2 meiocytes (n=20 cells scored in more than three independent experiments). Numbering indicates meiotic progression in minutes; t = 0 is just before spindle formation. Bars represent 5 μm.

Journal of Cell Science • Supplementary information

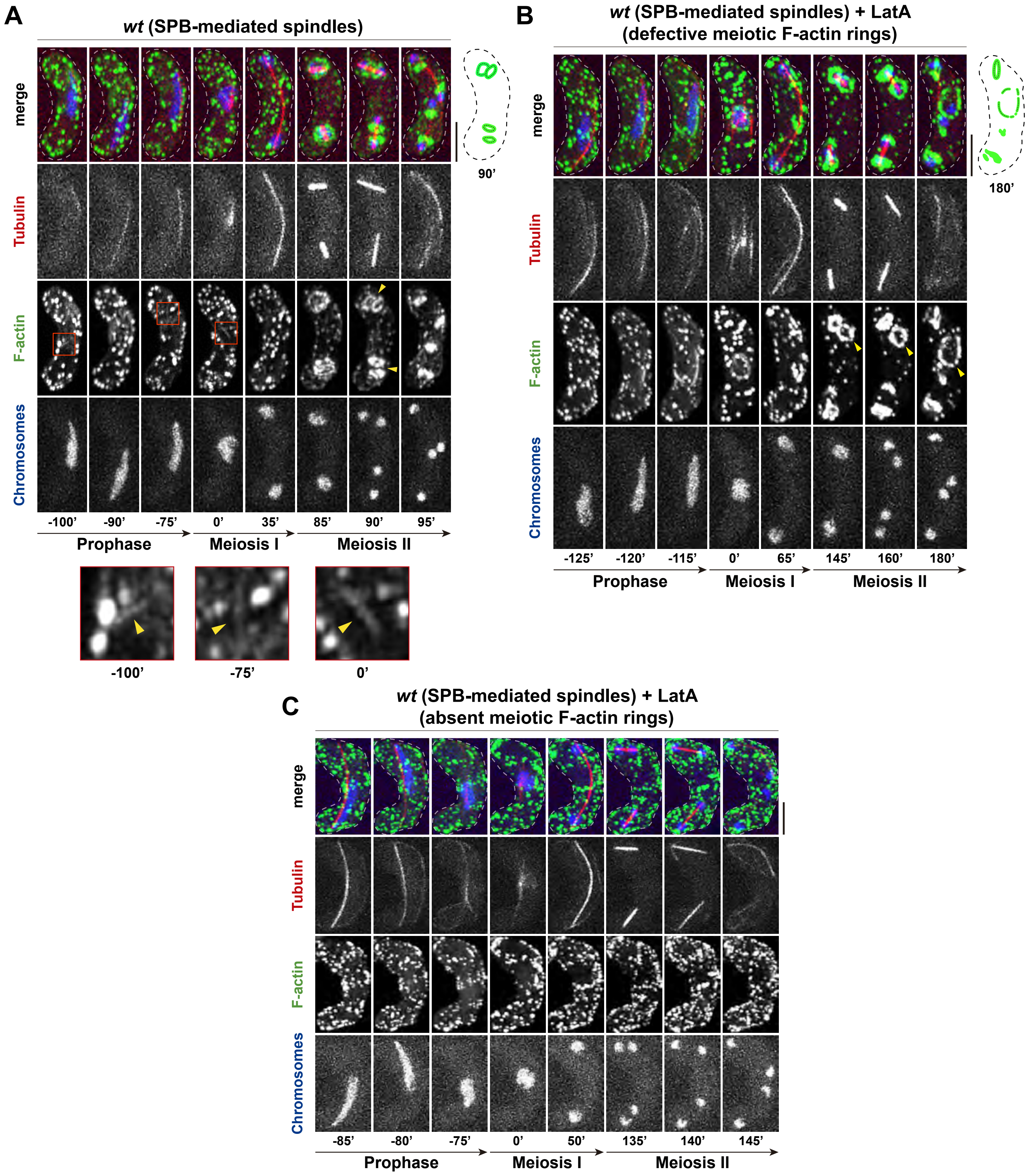

Fig. S4. (Complement to Figure 4). SPB-mediated meiotic spindle formation and behaviour are independent of the F-actin network in fission yeast meiosis. (A-C) Frames from films in meiosis. Numbers underneath represent time (in minutes) from MI onset. Scale bars represent 5 µm. F-actin networks is viewed via Lifeact-GFP, chromatin via histone H3 tagged at one of the two endogenous hht1+ loci, and tubulin via ectopically expressed mCherry-Atb2. (B-C) Behaviour of SPB-mediated and F-actin network upon addition of actin-depolymerizing drug Latrunculin A (LatA, 4 µM). Various effects of LatA on meiotic actin rings morphology/dynamics are shown in (B, 145' to 180') and (C, 135' to 145').

Journal of Cell Science • Supplementary information

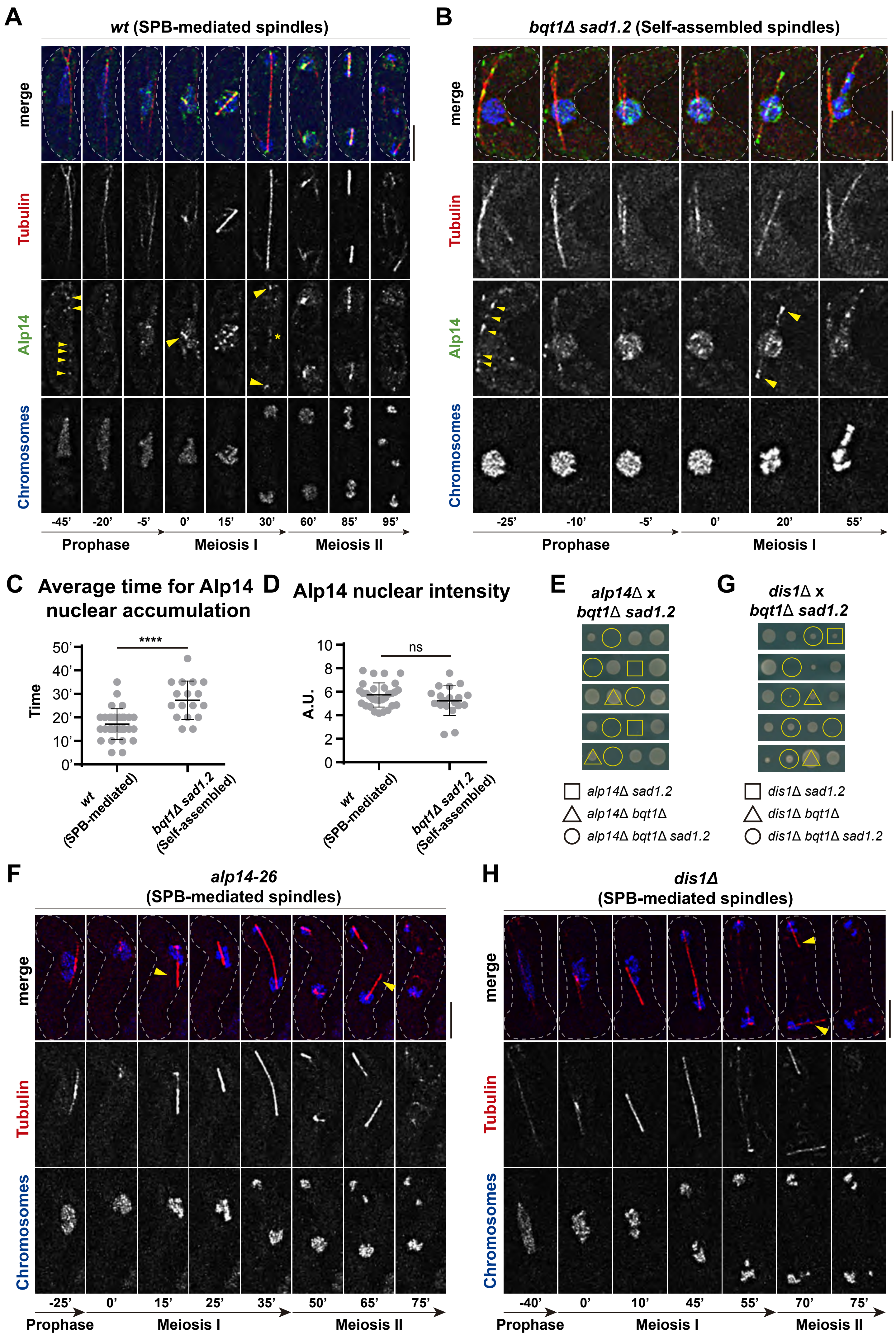

Journal of Cell Science • Supplementary information

Fig. S5. (Complement to Figure 6). Alp14/XMAP215 microtubule polymerase localizes to self-assembled spindles. (A-B) Frames from films of meiocytes carrying chromosomes and spindles tagged as in Figure 6 with endogenously GFP-tagged Alp14. Numbering indicates meiotic progression in minutes; t = 0 is just before spindle formation. Bars represent 5 µm. Yellow arrowheads indicate localization of Alp14 at oscillating microtubules in prophase (A, -45'), at the tubulin focus from which the spindle forms (A, 0') and at MI SPB-mediated spindle poles (A, 30'). Yellow asterisk indicates localization of Alp14 at the MI SPB-mediated spindle midzone (A, 30'). (B) Localization of Alp14 during meiosis in the *bqt1Δ sad1.2* background. Yellow arrowheads indicate localization of Alp14 at oscillating microtubules in prophase (-25') and at poles of the self-assembled spindle (20'). (C) Average time for Alp14 nuclear accumulation for SPB-mediated and self-assembled spindles in *wt* and *bqt1Δ sad1.2* backgrounds, respectively. t-test: ****, $P < 0.0001$). N = 30 for wt, N = 17 for *bqt1Δ sad1.2.* (D) Quantification of maximum nuclear fluorescence signal of Alp14. t-test: ns, $P > 0.05$. N = 30 for wt, N = 19 for *bqt1Δ sad1.2*. (C-D) Bars represent mean and standard deviation. (E) *bqt1*Δ *sad1.2 alp14*Δ cells present a synthetic lethality when spores germinate after tetrads dissection analysis. Spores were grown at 25ºC for 5 days. 52 asci were analysed with 94% of lethality for *bqt1*Δ *sad1.2 alp14*Δ. (F) Extra example to Figure 6B. (G) Spore analysis after tetrads dissection. Spores were grown at 25ºC for 5 days. 66 asci were analysed showing *bqt1*Δ *sad1.2 dis1*Δ 37% of lethality. (H) Extra example to Figure 6G. (F-H) Yellow arrowheads depict spindle behavior defects.

Journal of Cell Science • Supplementary information

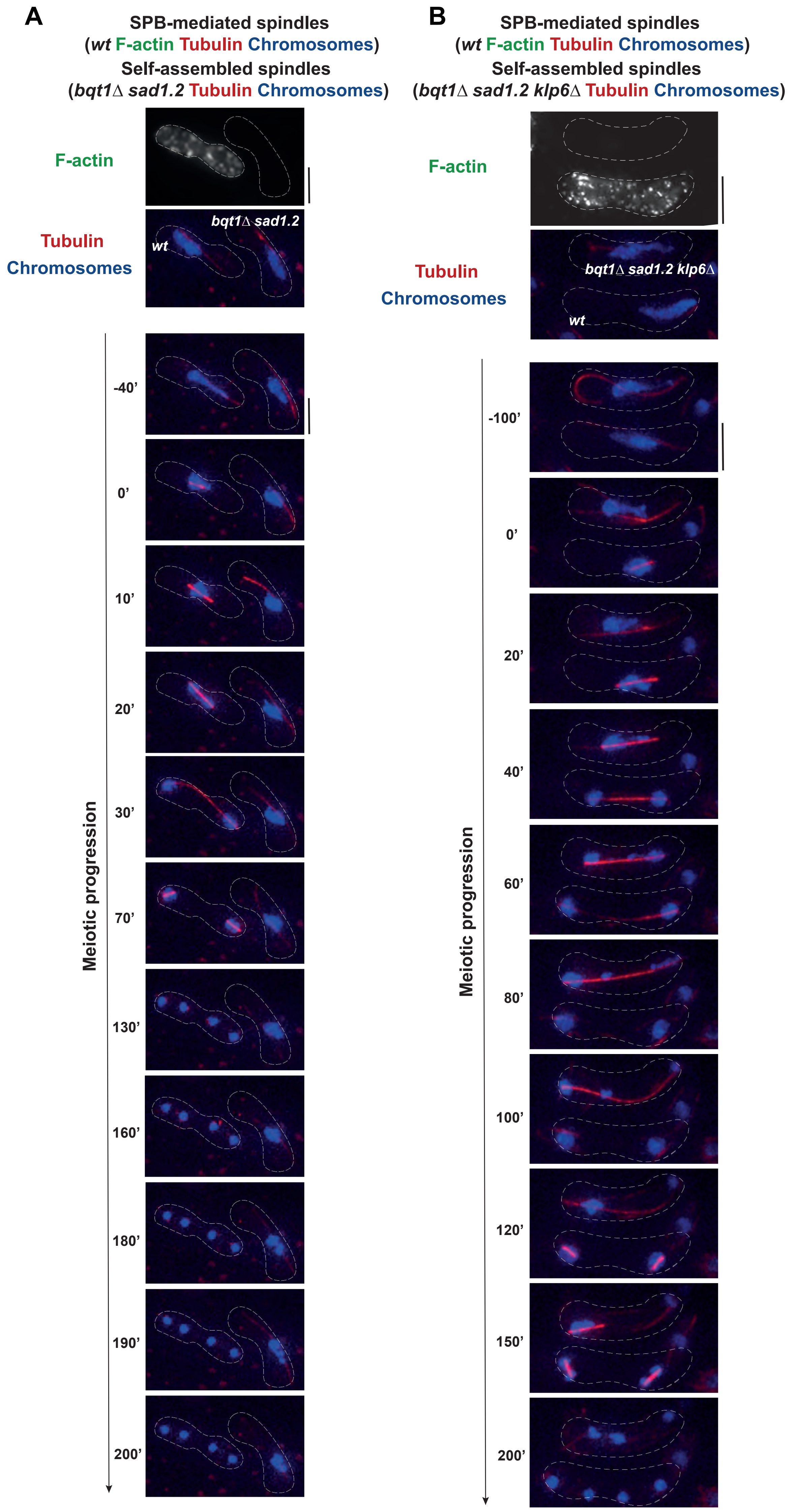

Fig. S6. (Complement to Figure 7). Elimination of Klp6 improves spindle self-assembly in *bqt1*Δ *sad1.2* cells. Frames from films of meiocytes carrying chromosomes and spindles tagged as in Figure 7. *wt* cells harbour Lifeact-GFP as a genotype marker. *wt* and *bqt1*Δ *sad1.2* meiocytes were patched on the same SPA plate and filmed in the same field. Numbering indicates meiotic progression in minutes; t = 0 is just before spindle formation of the *wt* cell. Bars represent 5 µm.

Journal of Cell Science • Supplementary information

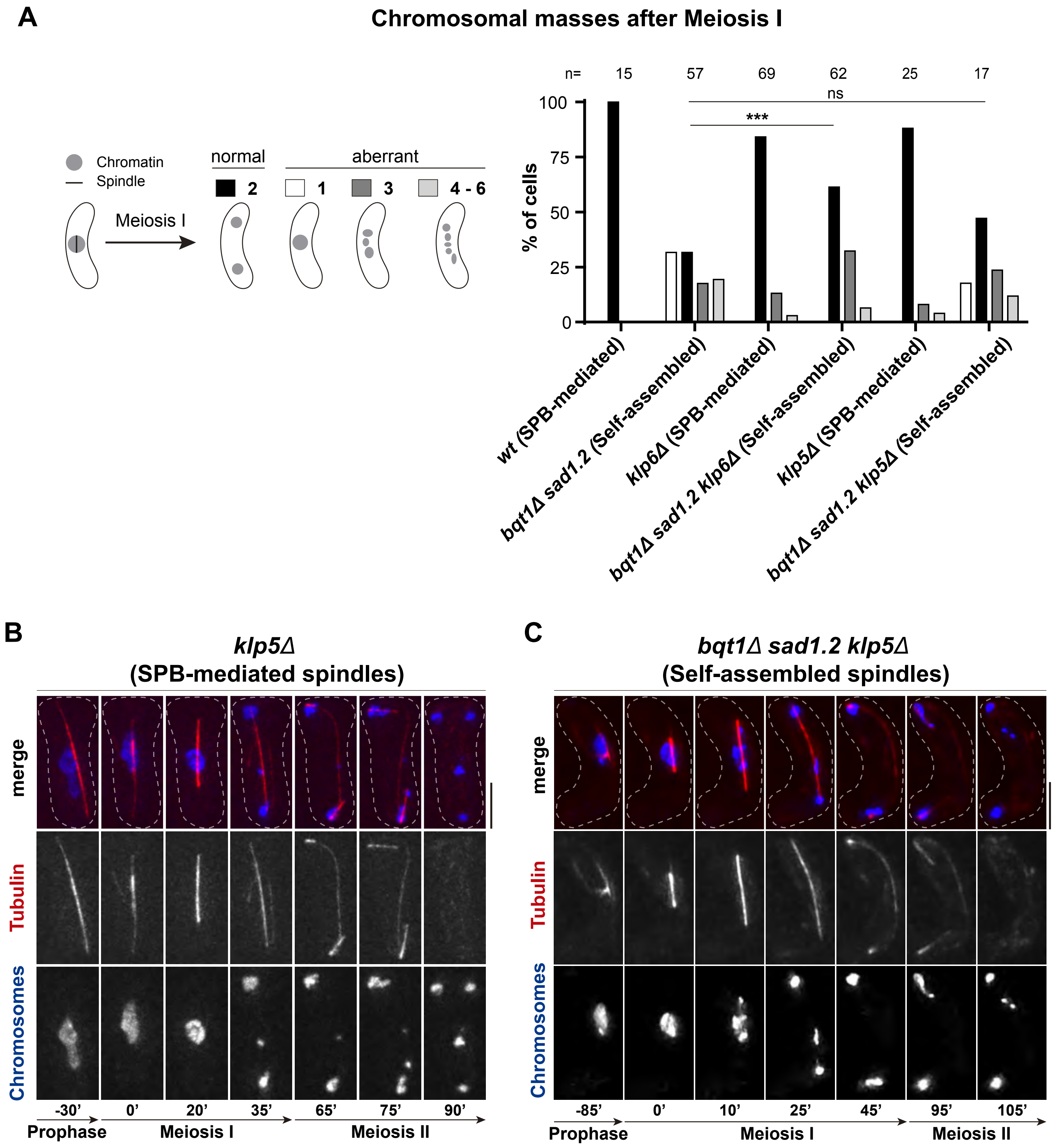

Fig. S7. (Complement to Figure 7). Elimination of Klp5 improves spindle self-assembly in *bqt1*Δ *sad1.2* cells. (A) Quantification of chromosome segregation fidelity of SPB-mediated and self-assembled spindles in presence and absence of *klp6*. Fisher's exact test for the category of two chromosomal masses after MI: *, P < 0.05; ***, P < 0.001; ****, P < 0.0001. n is the total number of cells scored from more than three independent experiments. (B-C) Frames from films of meiocytes carrying chromosomes and spindles tagged as in Figure 7. Numbering indicates meiotic progression in minutes; t = 0 is just before spindle formation. Bars represent 5 μm.

Journal of Cell Science • Supplementary information

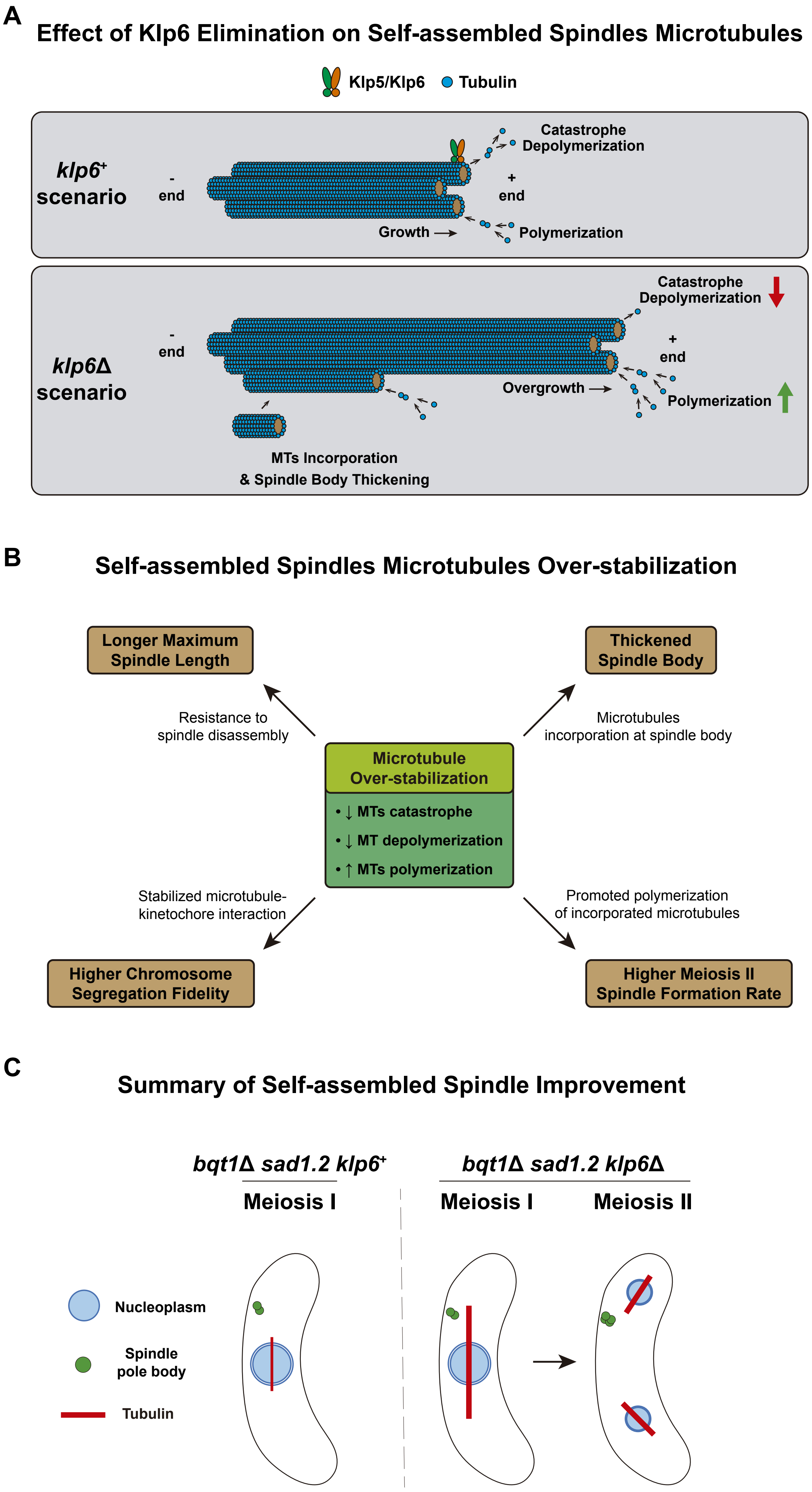

Fig. S8. (Complement to Figure 8) Schematic of proposed mechanisms of self-assembled spindle improvement by elimination of Klp6. (A) Speculated primary effects of elimination of microtubule-destabilizing activity of Klp5/Klp6 (klp6Δ setting) upon microtubule dynamics equilibrium. (B) Different pathways through which microtubule over-stabilization caused by Klp6 elimination could be responsible for self-assembled spindles improvement. (C) Representation of the global improvement of self-assembled spindle structure, formation and chromosome segregation fidelity by elimination of Klp6.

Table S1. Strains used in this study

Strain	Mating Type	Genotype	Origin
**AFA51**	h^{90}	*ade6-M210 ura4-D18 hht1-CFP:his3 Pnda3-mCherry-Atb2:aur1*	Pineda-Santaella et al., 2019
**AFA54**	h^{90}	*ade6-M210 ura4-D18 hht1-CFP:his3 Pnda3-mCherry-Atb2:aur1 sad1.T3S.S52P-13Myc-hphMX6 bqt1::hygMX6*	Pineda-Santaella et al., 2019
**AFA119**	h^{90}	*ade6-M210 hht1-CFP:his3 Pnda3-mCherry-Atb2:aur1 Pact1-LA-GFP:leu1+*	This study
**AFA206**	h^{+}	*his3-D1 hht1-CFP:his3+ Pnda3-mCherry-atb2:aur1 alp4-GFP:kanMX6*	This study
**AFA210**	h^{90}	*his3-D1 hht1-CFP:his3+ Pnda3-mCherry-atb2:aur1 alp4-GFP:kanMX6*	This study
**AFA219**	h^{90}	*hht1-CFP:his3+ Pnda3-mCherry-atb2:aur1 alp4-GFP:kanMX6 sad1.T3S.S52P-13Myc-hphMX6 bqt1::hygMX6*	This study
**AFA306**	h^{90}	*ade6-M210 ura4-D18 hht1-CFP:his3 Pnda3-mCherry-Atb2:aur1 ase1Δ::KanMX6*	This study
**AFA316**	h^{90}	*ade6-M210 ura4-D18 hht1-CFP:his3 Pnda3-mCherry-Atb2:aur1 sad1.T3S.S52P-13Myc-hphMX6 bqt1::hygMX6 ase1Δ::KanMX6*	This study
**AFA319**	h^{90}	*hht1-CFP:his3 Pnda3-mCherry-Atb2:aur1 sad1.T3S.S52P-13Myc-hphMX6 bqt1::hygMX6 Pact1-LA-GFP:leu1+*	This study
**AFA336**	h^{90}	*hht1-CFP:his3 Pnda3-mCherry-Atb2:aur1 alp14-GFP:Kan*	This study
**AFA339**	h^{90}	*his3-D1 hht1-CFP:his3 Pnda3-mCherry-Atb2:aur1 sad1.T3S.S52P-13Myc-hphMX6 bqt1::hygMX6 alp14-GFP:Kan*	This study
**AFA350**	h^{90}	*kanR-Palp4-GFP-pkl1 hht1-CFP:his3 aur1R-Pnda3-mCherry-atb2*	This study
**AFA406**	h^{90}	*kanR-Palp4-GFP-pkl1 hht1-CFP:his3 Pnda3-mCherry-Atb2:aur1 sad1.T3S.S52P-13Myc-hphMX6 bqt1::hygMX6*	This study
**AFA416**	h^{90}	*ade6-M210 hht1-CFP:his3+ Pnda3-mCherry-Atb2:aur1 sad1-GFP:HygMX6*	This study
**AFA437**	h^{90}	*ade6-M210 hht1-CFP:his3+ Pnda3-mCherry-atb2:aur1 bqt1::hygMX6 sad1.2-GFP:KanMX6*	This study
**AFA438**	h^{90}	*hht1-CFP:his3+ Pnda3-mCherry-Atb2:aur1 sad1.2-GFP:HygMX6*	This study
**AFA450**	h^{90}	*hht1-CFP:his3+ Pnda3-mCherry-atb2:aur1 klp9-GFP:KanMX6*	This study
**AFA467**	h^{90}	*hht1-CFP:his3 Pnda3-mCherry-Atb2:aur1 sad1.T3S.S52P-13Myc:hphMX6 bqt1::hygMX6 klp9-GFP*	This study
**AFA498**	h^{+}	*his3-D1 hht1-CFP:his3+ Pnda3-mCherry-atb2:aur1 alp4-GFP:kanMX6*	This study
**AFA503**	h^{+}	*kanR-Palp4-GFP-pkl1 hht1-CFP:his3 aur1R-Pnda3-mCherry-atb2*	This study
**AFA507**	h^{-}	*hht1-CFP:his3+ Pnda3-mCherry-atb2:aur1 sad1-mRFP:kan*	This study
**AFA509**	h^{-}	*hht1-CFP:his3+ Pnda3-mCherry-atb2:aur1 sid4-mCherry:NatMX6*	This study
**AFA528**	h^{-}	*sad1.2-mCherry:hygMX6 bqt1::NatMX6 alp4-GFP:kanMX6 hht1-CFP:his3 mCherry-atb2:aur1*	This study
**AFA530**	h^{+}	*sad1.2-mCherry:hygMX6 bqt1::NatMX6 alp4-GFP:kanMX6 hht1-CFP:his3 mCherry-atb2:aur1*	This study
**AFA531**	h^{+}	*sad1.2-mCherry:hygMX6 bqt1::natMX6 GFP-Pkl1:kanMX6 hht1-CFP:his3 mCherry-atb2:aur1*	This study
**AFA533**	h^{-}	*sad1.2-mCherry:hygMX6 bqt1::natMX6 GFP-Pkl1:kanMX6 hht1-CFP:his3 mCherry-atb2:aur1*	This study
**AFA534**	h^{+}	*sad1.2:natMX6 bqt1::hygMX6 alp4-GFP:kanMX6 hht1-CFP:his3+ Pnda3-mCherry-atb2:aur1*	This study
**AFA535**	h^{-}	*sad1.2:bleMX6 bqt1::hygMX6 sid4-mCherry:NatMX6 hht1-CFP:his3+ Pnda3-mCherry-atb2:aur1*	This study
**AFA543**	h^{+}	*sad1.2:natMX6 bqt1::hygMX6 kanR-Palp4-GFP-pkl1 hht1-CFP:his3 aur1R-Pnda3-mCherry-atb2*	This study
**AFA550**	h^{90}	*dis1::ura4+ sad1.2:hygMX6 bqt1::natMX6 hht1-CFP:his3+ mCherry-atb2:aur1*	This study
**AFA551**	h^{+}	*dis1::ura4+ hht1-CFP:his3+ mCherry-atb2:aur1*	This study
**AFA552**	h^{-}	*dis1::ura4+ hht1-CFP:his3+ mCherry-atb2:aur1*	This study
**AFA1431**	h^{90}	*ade6- hht1-CFP:his3 Pnda3-mCherry-Atb2:aur1 klp6::kanMX6*	This study
**AFA1437**	h^{90}	*hht1-CFP:his3 Pnda3-mCherry-Atb2:aur1 sad1.T3S.S52P::natMX6 bqt1::hygMX6 klp6::kanMX6*	This study

Journal of Cell Science • Supplementary information
