# Supplementary material for: Loss of kinesin-8 improves the robustness of the self-assembled spindle in Schizosaccharomyces pombe
Source: J Cell Sci. 2021 Aug 23;134(16):jcs253799. doi: 10.1242/jcs.253799 (PMC8435293; doi:10.1242/jcs.253799)
Supplement: Reviewer comments [file joces-134-253799_review_history.pdf]

Journal of Cell Science

Loss of kinesin-8 improves the robustness of the self-assembled spindle in *Schizosaccharomyces pombe*

Alberto Pineda-Santaella, Nazaret Fernández-Castillo, Alberto Jiménez-Martín, María del Carmen Macías-Cabeza, Ángela Sánchez-Gómez and Alfonso Fernández-Álvarez
DOI: 10.1242/jcs.253799

Editor: David Glover

Review timeline

Original submission:	2 September 2020
Editorial decision:	29 October 2020
First revision received:	18 May 2021
Accepted:	19 July 2021

Original submission

First decision letter

MS ID#: JOCES/2020/253799

MS TITLE: Loss of Kinesin-8 improves the robustness of the acentrosomal spindle

AUTHORS: Alberto Pineda-Santaella, Nazaret Fernandez-Castillo, Angela Sanchez-Gomez, and Alfonso Fernandez-Alvarez
ARTICLE TYPE: Research Article

We have now reached a decision on the above manuscript.

To see the reviewers' reports and a copy of this decision letter, please go to: https://submit-jcs.biologists.org and click on the 'Manuscripts with Decisions' queue in the Author Area. (Corresponding author only has access to reviews.)

As you will see, the reviewers raise a number of substantial criticisms that prevent me from accepting the paper at this stage. They suggest, however, that a revised version might prove acceptable, if you can address their concerns. If you think that you can deal satisfactorily with the criticisms on revision, I would be pleased to see a revised manuscript. We would then return it to the reviewers.

We are aware that you may be experiencing disruption to the normal running of your lab that makes experimental revisions challenging. If it would be helpful, we encourage you to contact us to discuss your revision in greater detail. Please send us a point-by-point response indicating where you are able to address concerns raised (either experimentally or by changes to the text) and where you will not be able to do so within the normal timeframe of a revision. We will then provide further guidance. Please also note that we are happy to extend revision timeframes as necessary.

Please ensure that you clearly highlight all changes made in the revised manuscript. Please avoid using 'Tracked changes' in Word files as these are lost in PDF conversion.

I should be grateful if you would also provide a point-by-point response detailing how you have dealt with the points raised by the reviewers in the 'Response to Reviewers' box. Please attend to

© 2021. Published by The Company of Biologists under the terms of the Creative Commons Attribution License (https://creativecommons.org/licenses/by/4.0/).

all of the reviewers' comments. If you do not agree with any of their criticisms or suggestions please explain clearly why this is so.

Reviewer 1

Advance summary and potential significance to field

The manuscript continues the analysis of spindles formed in the apparent absence of MTOCs in fission yeast meiosis that the authors recently published in Chromosoma. In this manuscript they provide further evidence that the yeast SPD is not involved by studying the localisation of Sad1. They also extend the analysis of these partially functional spindles by demonstrating that Ase1 is required for their integrity, that they share the same structural polarity with SPB-dependent spindles and that Alp1, which functions to polymerise microtubules, is located at these spindles. Unfortunately, they do not genetically indicate a requirement for Alp1. They also report that, unlike oocyte self-assembling spindles, the actin network is not involved. Finally loss of Klp6, a kinesin known to destabilise microtubules, is shown to increase the robustness and functionality of the self-assembling spindles. The work adds confidence, but does not categorically demonstrate, that these self-assembling spindles are formed without input from the MTOC and adds perhaps expected observations of how such spindles, which can segregate chromosomes, are influenced by various known factors. Part of the claimed impact is that this is a good model for oocytes, but this is not so clear to me as there are differences, such as the lack of involvement of Actin fibres and it is likely that evolution has optimised stability of oocyte spindles, limiting the insight gained from removing factors like Klp6.

Comments for the author

The manuscript would benefit from editing for English usage. The data are generally well presented and support the claims made.
In figure 1 it should be indicated clearly what percentage of Asci undergo spindle formation.
To establish a function for Ase1 would strengthen the conclusion that it is involved. Possibly, this could be achieved with the use of an AID system. Similarly, AID could be used to robustly demonstrate that the gamma-tubulin complex is not involved. Such a conclusion is hard to make strongly on the basis of fluorescence microscopy.
line 229-301. The explanation of the data is not very clear, please include the fourth percentage.
line 401-412. I did not feel the speculation in this paragraph added anything useful.
Figure 8 could be supplementary.
The figure legends draw conclusions from the data, rather than describe the data. They should be rewritten be describe the data and the conclusions should be contextualised by the main text.

Reviewer 2

Advance summary and potential significance to field

In this manuscript, Pineda-Santaella et al investigate the assembly of microtubule arrays that form during fission yeast sexual differentiation in particular genetic backgrounds. They refer to these arrays as Self assembled spindles. After describing the arrays in a bqt1D sad1.2 mutant background they continue their study in an undefined background/context in which they introduce additional markers and mutations to determine the behaviour of some well characterised mitotic spindle components and the F-actin cytoskeleton.

The key observation is that the removal of kinesin 8 improves the robustness of these microtubule arrays. As such, if all these structures are indeed self-assembled spindles and further controls are conducted, this study marks a significant advance in our understanding of meiotic spindle function in a very popular model organism. This manuscript will therefore be suitable for publication in Journal of Cell Science should a number of concerns be addressed.

Comments for the author

Major points General points:

© 2021. Published by The Company of Biologists under the terms of the Creative Commons Attribution License (https://creativecommons.org/licenses/by/4.0/).

It is unclear throughout the study how the authors are differentiating the prophase microtubule array from the array they call a self-assembled spindle. In a wild type context, it is possible to use the morphology of the nucleus as the horsetail shape defines prophase and, once conjugation and karyogamy are complete, any other, more compact chromatin configuration defines the stages of meiosis. However, when the chromosomes are not being efficiently grabbed by the SPB, they do not form a horsetail and so the chromatin may form a similar morphology to the chromatin when the chromosomes are not being pulled efficiently by the spindle. Thus, a prophase bundle whose horsetail association is poor will resemble an intranuclear self-assembling spindle that is accompanied by an amorphous mass of chromatin whose association with the microtubules is poor. It is therefore important to incorporate a marker for the nucleus, such as an NLS fluorescent protein construct, or the nuclear envelope, in order to define the perimeter of the nucleus and so be able to definitively state that the structures being studied are indeed intranuclear structures. This demonstration that the bundle in question sits within the nucleus, and so is indeed a self-assembled spindle needs to be done in every part of the study.

It is unclear what the genotype is of the cells used to show the self-assembled spindles. In each section the behaviour of the wild type spindle is discussed and then the self-assembled spindle. I therefore consulted the figure legends to see whether my assumption that each time a self-assembled spindle is referred to it is indeed a bqt4 sad1.2 mutant, but could find no confirmation. This needs to be addressed, because one gets the impression in some places that these self-assembled spindles are a variant that is assembling in a wild type setting. It is therefore really important to re-read the figure legends and make them far more comprehensive, giving the reader the full genotype and labelling the figures accordingly, so it is clear when we are looking at wild type cells and when it is a given mutant. Similarly, with the main text. At present the labelling of “self assembled spindles” is not providing sufficient clarity.

There are several places where a greater degree of scholarship would be beneficial.

• The electron microscopy work from Dick McIntosh’s lab is superb, but so is the EM work of Carl Robinow.
Robinow was one of the great scholars of fungal cell biology of his era – it was his work that coined the term “horsetail movement” (Robinow CF, 1977 Genetics 87:491-497). The McCully and Robinow study is a master class in conventional fixation of fission yeast (McCully, K. Robinow, CF (1971) J Cell Sci. 9:475-507). It shows the interphase SPB (they call it the kinetochore equivalent (KCE)) on the cytoplasmic face of the nuclear envelope, the insertion into the nuclear envelope and extrusion in late spindles. Kanbe and Tanka (Kanbe and Tanaka 1986 J Cell Sci. 80:253-268) who introduced freeze substitution via plunge freezing to fission yeast analyses also show this before the high-pressure freezing approach of the Ding study in the McIntosh lab. It would therefore seem only fair to refer to the Robinow and Tanaka contributions alongside the landmark McIntosh study.

• It is surprising that the authors state that “…..its (the actin cytoskeleton) role for meiotic spindle formation and behaviour has not been yet disclosed.” The study by Petersen et al. 1998, describes the F-actin cytoskeleton throughout sexual differentiation from conjugation through meiosis and uses latrunculin A to determine the contribution of the actin cytoskeleton to fusion, karyogamy and genome segregation. They show that full actin dissolution reduces the number of 4 nuclei cells in their synchronised system from 32% to 13%. Thus, the meiosis is still going on in the absence of actin. (Petersen et al. F-actin distribution and function during sexual differentiation in Schizosaccharomyce pombe. J Cell Sci. 111 867-876). The authors appear to have missed this study. This section on F-actin should therefore be amended accordingly. The authors may wish to consider whether it would be good to elaborate on what the actin rings are at the point in the results where they are referred to. This clarity may help the general fission yeast audience, because most fission yeast scholars think of actin rings as cytokinetic actin rings, however, as the authors point out in the discussion, during meiosis II, the rings represent the leading edges of the forespore membrane at which vesicle fusion is extending the membrane to encompass the nucleus within the spore.

• It is stated that “in fission yeast, the microtubule polymerase XMAP215 family is represented by the well-
characterised Alp14 protein (Al-Bassam et al. 2012). This is incorrect. There are two members of the family in fission yeast – Dis1 and Alp14. Dis1 is required for growth at low temperature, Alp14 at high temperatures.

© 2021. Published by The Company of Biologists under the terms of the Creative Commons Attribution License (https://creativecommons.org/licenses/by/4.0/).

Enhancing dis1 levels suppresses the temperature sensitive phenotype of alp14.D and vice versa. Thus, there are two, redundant members of the same family and one may assume that the different temperature sensitivities of the knockouts reflect different contributions to spindle function, that are differentially required when microtubule dynamics are reduced (Dis1), or enhanced (Alp14). It is therefore misleading to attribute all chTOG activity to Alp14. The authors may therefore want to consider whether the behviour of the second chTOG family member merits analysis in their revised study. It would certainly seem to be appropriate to do so.
For figure 3 it is important to show co-localisation of Sad1 and Pkl1 and co-localisation of a core SPB marker such as Sid4 and Pkl1 to categorically show that the Pkl1 foci are at the end of microtubule bundles and not SPB remnants.

Lines 230 -232 "…pointing that these dots correspond to y-tubulin complex association to the uninserted dislodged SPBs." There is a need to show co-staining with an SPB marker here to be able to say this.
Line 291 it is important to show and quantitate the differential level of intensity in mixed cultures of the klp6 wild type and klp6 side by side in the same field of view. Then the comparison is truly meaningful. There is always the possibility that differences taken on different sessions on a microscope arise from fluctuations in illumination, capture, or processing. The two lines within the same field of view would maybe differentiated via staining with a cell surface binding lectin, or the expression of a fluorescent marker in one strain but not the other.
Minor points On line 134 it is stated ", which consists of a dismantling of the whole spindle body (Fig. 2….". The similarity between spindle body and spindle pole body is a bit confusing – the authors may prefer to change the wording – perhaps spindle structure?
The gap in the tubulin signal in Figure 2D is not particularly convincing because the entire tubulin structure is comprised of a series of puncta, rather than a linear continuum. It would therefore be good to include some further examples in supplementary data.
Figure 7E – the bars seem to be misaligned in the formation of the pdf document for submission.

Reviewer 3

Advance summary and potential significance to field

Using live cell microscopy, the authours described the fission yeast double-mutant sad1.2 bqtΔ, where, independent of the SPB, a spindle-like structure can form and can poorly segregate the chromosome at meiosis. They showed that the SPB-independent spindle-like structure has structural similarities to bona fide spindles. Thus, they proposed that the spindle-like structures of fission yeast can be viewed as acentrosomal meiotic spindles, present in other organisms. They then showed that the absence of kinesin-8 in these spindle-like structures enhances their ability to properly segregate chromosomes, suggesting that the same could be true of acentrosomal spindles.

Comments for the author

I cannot currently recommend publication of the present work in J Cell Sci.
There are major concerns:
1) The Abstract effectively states that acentrosomal spindles causes high rate of aneuploidy, which is a strong over-statement. Then the authours claim that the SPB-independent spindle-like structures of fission yeast can be a proxy for studying bonafide acentrosomal spindles and chromosome segregation. This again, is a strong over-statement and simplification.

2) The description of the fission yeast double-mutant sad1.2 bqtΔ and the SPB-independent spindle-like structures, has been previously reported by the same authors (Pineda-Santaella 2019 Chromosoma). The current work extends the analysis to the function of kinesin-8 – its absence make the spindle-like structure more efficiency in segregating chromosomes. While this suggests a potential role for kinesin-8 in bona fide acentrosomal spindles the authours have not validated this.

3) In the SPB-independent spindle-like structures, chromosomes are clearly not properly segregated. However, no markers for kinetochores or centromeres were used in the current study, preventing proper interpretation/conclusion of actual kinetochore-dependent chromosome

© 2021. Published by The Company of Biologists under the terms of the Creative Commons Attribution License (https://creativecommons.org/licenses/by/4.0/).

segregation or passive chromosome separation/fragmentation in these mutants. Again, claiming the SPB-independent spindle-like structure as bonafide acentrosomal spindles is an over-statement. I would be more positive if points 2 and 3 were experimentally addressed.

First revision

Author response to reviewers' comments

Dear Editor,

We hereby submit a revised version of the manuscript JOCES/2020/253799, entitled 'Loss of Kinesin-8 improves the robustness of the acentrosomal spindle'. We are grateful for the comments made by the reviewers and thank them for their constructive suggestions. We have addressed all the reviewers' comments and in doing so, have clarified and reinforced the new observations we are presenting.

Here, we respond to each of the reviewers' comments in turn:

Reviewer 1 Advance Summary and Potential Significance to Field:
The manuscript continues the analysis of spindles formed in the apparent absence of MTOCs in fission yeast meiosis that the authors recently published in Chromosoma. In this manuscript they provide further evidence that the yeast SPB is not involved by studying the localisation of Sad1. They also extend the analysis of these partially functional spindles by demonstrating that Ase1 is required for their integrity, that they share the same structural polarity with SPB-dependent spindles and that Alp14, which functions to polymerise microtubules, is located at these spindles. Unfortunately, they do not genetically indicate a requirement for Alp14.

Thanks for the comment, we have analyzed the requirement of Alp14 for the formation of the self-assembled spindle and the results are shown in a new Figure 6. As also demonstrated in Figure S5E by tetrad dissection, the triple mutation *bqt1Δ sad1.2 alp14Δ* compromises cell viability during vegetative growth. For this reason, we have worked with the *alp14-26* thermosensitive allele at semipermissive temperature 28ºC, in our hands, the optimal temperature for meiotic progression in *wt* fission yeast. We found that Alp14 is crucial for the normal formation of self- assembling spindles (see quantification in Figure 6F, please).). All these results have been added and discussed in the revised version of the paper along with the study of the other member of the XMAP215 family, Dis1 (new panels G and H in Figure 6, and quantification in Figure 6F).

They also report that, unlike oocyte self-assembling spindles, the actin network is not involved. Finally, loss of Klp6, a kinesin known to destabilise microtubules, is shown to increase the robustness and functionality of the self-assembling spindles. The work adds confidence, but does not categorically demonstrate, that these self-assembling spindles are formed without input from the MTOC and adds perhaps expected observations of how such spindles, which can segregate chromosomes, are influenced by various known factors.

We want to mention that in a previous work by Julie Cooper's laboratory (Fernandez-Alvarez, et al., Dev Cell, 2016), we observed by electron microscopy that *bqt1Δ* cells present defects in the insertion of the SPB into the nuclear envelope (NE), the complete SPB is found in the cytoplasm away from the nucleus (see Figure 1I in Fernandez-Alvarez, et al., Dev Cell, 2016 please). We agree that it is difficult to categorically rule out any input from the SPB on the type of self-assembling spindles that we are observing, but the analysis of the location of Sad1 (see Figure 1 in the revised version of the manuscript) together with the reported analysis of the locations of Pcp1, Sid4 and Cut12 in *bqt1Δ sad1.2* cells (see Figure 2 in Pineda-Santaella & Fernandez-Alvarez, Chromosoma, 2019) strongly suggest the independence of the SPB in the assembly of this type of spindles. To reinforce this important premise of our work, we have enriched the revised version of the article with two new experiments as suggested by the reviewer 2: to evaluate together the

© 2021. Published by The Company of Biologists under the terms of the Creative Commons Attribution License (https://creativecommons.org/licenses/by/4.0/).

location of Alp4 and Pkl1 with Sad1 (LINC) and Sid4 (SPB) in the context of the self-assembled spindle configuration. We have performed these experiments which are shown in Figure S3 (Pkl1) and Figure 5C-5F (Alp4). These analyzes reinforce the idea that the self-assembled spindles in the *bqt1Δ sad1.2* meiocytes are independent of SPB and LINC, which, in our opinion, is a type of acentrosomal spindle.

Part of the claimed impact is that this is a good model for oocytes, but this is not so clear to me as there are differences, such as the lack of involvement of Actin fibres and it is likely that evolution has optimised stability of oocyte spindles, limiting the insight gained from removing factors like Klp6.

Thank you for giving us the opportunity to clarify this point. We apologize if in some parts of the previous version of the manuscript some ideas were so pretentious in the sense of trying to use our system as model for oocytes. We would like to clarify that our main goal is to understand the molecular mechanisms behind spindle self-assembly, a self-organizing system conserved throughout evolution. We believe that the system we have designed in S. pombe is competent to explore the molecular mechanisms behind this type of self-organizing microtubules around chromosomes. In our opinion, we consider this fission yeast system to be a useful platform to identify and study the conserved mechanisms and key factors that control the formation of self-assembling spindles during meiosis. However, while we also think that some of the observations might be interesting to test on the acentrosomal spindle of mammalian oocytes, this is not the main goal of our work and we apologize for this confusion. We have rewritten the manuscript to highlight the interest of the self-assembling microtubule mechanism per se beyond the possible implications for female meiosis.

Reviewer 1 Comments for the Author:
-The manuscript would benefit from editing for English usage. The data are generally well presented and support the claims made.

Thanks for the suggestion, the revised manuscript has been now reviewed by a professional native English-speaking editorial service.

-In figure 1 it should be indicated clearly what percentage of Asci undergo spindle formation.

We have added a new panel to Figure 1 that includes this quantification (see Figure 1C)

-To establish a function for Ase1 would strengthen the conclusion that it is involved. Possibly, this could be achieved with the use of an AID system. Similarly, AID could be used to robustly demonstrate that the gamma-tubulin complex is not involved. Such a conclusion is hard to make strongly on the basis of fluorescence microscopy.

Thanks for the suggestion. We would like to clarify three limitations in our methodology: i) the use of drugs such as auxin or latrunculin A (as in Figure 4 and Figure S4) must be added after karyogamy, because, in our hands, only the addition of DMSO in *wt* meiotic cells before karyogamy disturbs nuclear fusion; ii) We tried to use the AID system to degrade Sad1 in the past, but this strategy did not work faithfully in our hands, there is probably not enough time to inactivate the protein before meiosis I ; iii) the optimum temperature to film meiosis in S. pombe is between 23 and 32ºC, since higher temperatures can affect karyogamy and meiotic progression.
For all these reasons, we are inclined to use the Ase1 deletion and, because Alp4 is essential for viability, the thermo-sensitive allele *alp4-1891*, but only in the temperature range between 23 to 32ºC.

In the context of the implications of Ase1 for spindle self-assembly, to better substantiate the observations of the *ase1Δ* phenotypes shown in Figure 2, we show a further quantification of the effect of the Ase1 deletion on spindle behavior. We now present that the loss of Ase1 shortens the maximum length of the self-assembled and SPB-mediated spindles (see Figure 2F). Furthermore, we have added an additional example of the breakage of self-assembled spindles structure by the removal of Ase1 to reinforce our arguments (Figure S2). In our opinion, these new data confirm the functional relevance of Ase1 in the role of maintaining the structural integrity of both types of spindles in meiosis, as previously described for SPB-mediated meitotic spindles

© 2021. Published by The Company of Biologists under the terms of the Creative Commons Attribution License (https://creativecommons.org/licenses/by/4.0/).

(Zheng et al., 2020).

Regarding Alp4, we attempted to inactivate the gamma-tubulin complex specifically during meiotic prophase using a thermo-sensitive allele *alp4-1891*. Unfortunately, we were unable to achieve Alp4 inactivation with this strategy, with ~ 100% of the *alp4-1891* control meiocytes capable of forming SPB-dependent spindles after meiosis indiction at 32ºC and later meiosis filmation also at 32ºC (see below, see the pdf "response to reviews" in Supplementary Information please).

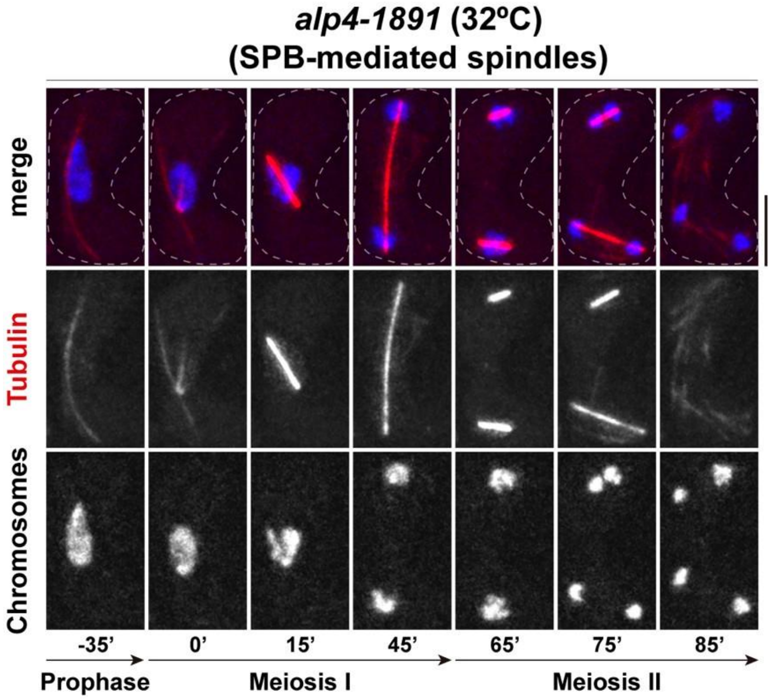

Using this thermo-sensitive allele, although we observed mitotic growth defects at semipermissive temperature (32ºC, see spot test below, see the pdf "response to reviews" in Supplementary Information please), the allele is more stable in meiosis where it might require temperatures above 32ºC and probably more time to inactivate.

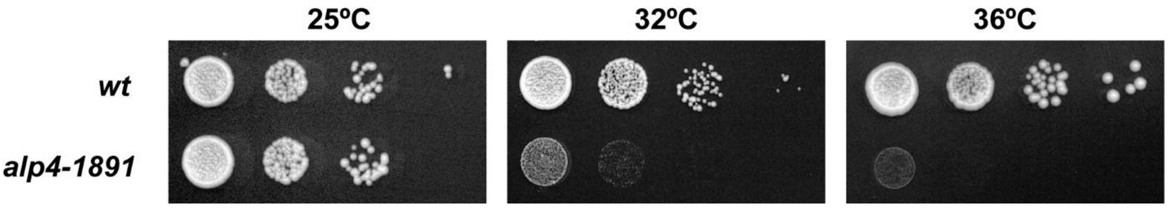

Due to these technical problems, we have rewritten this epigraph of the paper which now focuses on the location of Alp4 during self-assembly spindle formation compared to SPB-dependent spindles. We have also carried out new experiments but using Sid4 and Sad1, as SPB and LINC markers, respectively, to reinforce the idea that Alp4 seems to be absent from the poles of the self-assembled spindles.

-Line 229-301. The explanation of the data is not very clear, please include the fourth percentage.

As we said before, we have completed the Figure 5 with new experiments, where we have performed the analysis of Sid4 (SPB) and Sad1.2 (LINC) together with Alp4-GFP. Therefore, we can more easily visualize that Alp4-GFP is located in the SPBs and appears to be absent from the poles of the self-assembled spindles. We have included the number of cells analysed and the percentage of cells showing this phenotype in Figure Lengend 5.

© 2021. Published by The Company of Biologists under the terms of the Creative Commons Attribution License (https://creativecommons.org/licenses/by/4.0/).

-Line 401-412. I did not feel the speculation in this paragraph added anything useful.

Thanks for the suggestion. We have generated a more concise and less speculative version of this paragraph about the Alp4 location in the revised version of the document.

-Figure 8 could be supplementary.

We have moved it to Figure S8. Thanks for the suggestion.

-The figure legends draw conclusions from the data, rather than describe the data. They should be rewritten be describe the data and the conclusions should be contextualised by the main text.

We apologize for this formatting error. We have rewritten the figure legends based on your suggestion in a more concise way. Thanks.

Reviewer 2 Advance Summary and Potential Significance to Field:
In this manuscript, Pineda-Santaella et al investigate the assembly of microtubule arrays that form during fission yeast sexual differentiation in particular genetic backgrounds. They refer to these arrays as Self-assembled spindles. After describing the arrays in a bqt1D sad1.2 mutant background they continue their study in an undefined background/context in which they introduce additional markers and mutations to determine the behaviour of some well characterised mitotic spindle components and the F-actin cytoskeleton.

The key observation is that the removal of kinesin 8 improves the robustness of these microtubule arrays. As such, if all these structures are indeed self-assembled spindles and further controls are conducted, this study marks a significant advance in our understanding of meiotic spindle function in a very popular model organism. This manuscript will therefore be suitable for publication in Journal of Cell Science should a number of concerns be addressed.

Reviewer 2 Comments for the Author: Major points
General points:
It is unclear throughout the study how the authors are differentiating the prophase microtubule array from the array they call a self-assembled spindle. In a wild type context, it is possible to use the morphology of the nucleus as the horsetail shape defines prophase and, once conjugation and karyogamy are complete, any other, more compact chromatin configuration defines the stages of meiosis. However, when the chromosomes are not being efficiently grabbed by the SPB, they do not form a horsetail and so the chromatin may form a similar morphology to the chromatin when the chromosomes are not being pulled efficiently by the spindle. Thus, a prophase bundle whose horsetail association is poor will resemble an intranuclear self-assembling spindle that is accompanied by an amorphous mass of chromatin whose association with the microtubules is poor. It is therefore important to incorporate a marker for the nucleus, such as an NLS fluorescent protein construct, or the nuclear envelope, in order to define the perimeter of the nucleus and so be able to definitively state that the structures being studied are indeed intranuclear structures. This demonstration that the bundle in question sits within the nucleus, and so is indeed a self-assembled spindle, needs to be done in every part of the study.

We would like to clarify that in our previous work (Pineda-Santaella & Fernandez-Alvarez, Chromosoma 2019) we verified that the observed microtubule array is a nuclear event by using a nuclear membrane marker (Ish1-GFP, see Figure 3 in Pineda-Santaella & Fernandez-Alvarez, Chromosoma 2019, please), in this experiment, we can see how the nuclear envelope (NE) is pushed from the nucleoplam as a consequence of the spindle extension, even when the SPBs are clearly dislodged from the NE.

There are three important characteristics to differentiate between prophase microtubules and self-assembled spindles: i) dynamics; ii) polarity; iii) form around chromosomes after condensation.

i) The dynamics of self-assembled spindles is similar to that of canonical spindles mediated by the SPB: i) nucleation, ii) extension and iii) disassembly. During our time course analysis we take pictures every 5 minutes that allow us to identify all these stages. For this reason, it is relatively easy to identify and follow the formation of self-assemblied spindles.

© 2021. Published by The Company of Biologists under the terms of the Creative Commons Attribution License (https://creativecommons.org/licenses/by/4.0/).

ii) In the revised version of the article, we have added to the Figure 3 the location of Klp9- GFP (Figure 3B & 3D) along with the previously studied Pkl1-GFP to reinforce the arguments in favor of self-assembled spindles behaving like an SPB-dependent spindle in terms of polarity. Therefore, the location of both proteins on the self-assembled spindles is similar to that of the SPB- dependent spindles and clearly differs from prophase microtubule arrays (see Figure 3, please).

iii) As a criterion to define the reference time point (0 '), we use the frame just before the appearance of the microtubule arrangement that co-localizes with the condensed chromosomes, chromosomal condensation is easily recognizable and specific to meiotic initiation.

We believe that all of our data, globally, builds sufficient confidence for our conclusions. We agree with the reviewer that a nuclear membrane marker could ideally be used, but the fact that we use green, red and cyan in most experiments makes it difficult to use this marker for all experiments.

It is unclear what the genotype is of the cells used to show the self-assembled spindles. In each section the behaviour of the wild type spindle is discussed and then the self-assembled spindle. I therefore consulted the figure legends to see whether my assumption that each time a self-assembled spindle is referred to it is indeed a bqt4 sad1.2 mutant, but could find no confirmation. This needs to be addressed, because one gets the impression in some places that these self-assembled spindles are a variant that is assembling in a wild type setting. It is therefore really important to re-read the figure legends and make them far more comprehensive, giving the reader the full genotype and labelling the figures accordingly, so it is clear when we are looking at wild type cells and when it is a given mutant. Similarly, with the main text. At present the labelling of "self assembled spindles" is not providing sufficient clarity.

We apologize for the confusion. Self-assembled spindles are always associated with *bqt1Δ sad1.2* as they never occur in *wt* settings, but we agree that it is confusing. We have added the genotype in all figures and figure legends throughout the manuscript. Thanks for the suggestion.

There are several places where a greater degree of scholarship would be beneficial.
•The electron microscopy work from Dick McIntosh's lab is superb, but so is the EM work of Carl Robinow. Robinow was one of the great scholars of fungal cell biology of his era - it was his work that coined the term "horsetail movement" (Robinow CF, 1977 Genetics 87:491-497). The McCully and Robinow study is a master class in conventional fixation of fission yeast (McCully, K. Robinow, CF (1971) J Cell Sci. 9:475- 507). It shows the interphase SPB (they call it the kinetochore equivalent (KCE)) on the cytoplasmic face of the nuclear envelope, the insertion into the nuclear envelope and extrusion in late spindles. Kanbe and Tanka (Kanbe and Tanaka 1986 J Cell Sci. 80:253-268) who introduced freeze substitution via plunge freezing to fission yeast analyses also show this before the high-pressure freezing approach of the Ding study in the McIntosh lab. It would therefore seem only fair to refer to the Robinow and Tanaka contributions alongside thelandmark McIntosh study.

We apologize for not including all these works in the previous version of the article. We thank the reviewer for suggestions. We have included them in the revised version of the document.

•It is surprising that the authors state that "…..its (the actin cytoskeleton) role for meiotic spindle formation and behaviour has not been yet disclosed." The study by Petersen et al. 1998, describes the F-actin cytoskeleton throughout sexual differentiation from conjugation through meiosis and uses latrunculin A to determine the contribution of the actin cytoskeleton to fusion, karyogamy and genome segregation.
They show that full actin dissolution reduces the number of 4 nuclei cells in their synchronised system from 32% to 13%. Thus, the meiosis is still going on in the absence of actin. (Petersen et al. F-actin distribution and function during sexual differentiation in Schizosaccharomyce pombe. J Cell Sci. 111 867-876). The authors appear to have missed this study.

Thanks for the comment. We apologize for missing this reference. We fully agree with the reviewer that the work by Petersen et al. 1998 should be mentioned in our manuscript, which has been added to the revised version of the paper. Thanks again.

© 2021. Published by The Company of Biologists under the terms of the Creative Commons Attribution License (https://creativecommons.org/licenses/by/4.0/).

This section on F-actin should therefore be amended accordingly. The authors may wish to consider whether it would be good to elaborate on what the actin rings are at the point in the results where they are referred to.

This clarity may help the general fission yeast audience, because most fission yeast scholars think of actin rings as cytokinetic actin rings, however, as the authors point out in the discussion, during meiosis II, the rings represent the leading edges of the forespore membrane at which vesicle fusion is extending the membrane to encompass the nucleus within the spore.

Thanks for the suggestion, we have added "meiotic actin rings" in Figure 4 and Figure S4 and in the Results and Discussion sections to avoid confusion with actin cytokinesis rings.

•It is stated that "in fission yeast, the microtubule polymerase XMAP215 family is represented by the well- characterised Alp14 protein (Al-Bassam et al. 2012). This is incorrect. There are two members of the family in fission yeast - Dis1 and Alp14. Dis1 is required for growth at low temperature, Alp14 at high temperatures. Enhancing dis1 levels suppresses the temperature sensitive phenotype of alp14.D and vice versa. Thus, there are two, redundant members of the same family and one may assume that the different temperature sensitivities of the knockouts reflect different contributions to spindle function, that are differentially required when microtubule dynamics are reduced (Dis1), or enhanced (Alp14). It is therefore misleading to attribute all chTOG activity to Alp14. The authors may therefore want to consider whether the behviour of the second chTOG family member merits analysis in their revised study. It would certainly seem to be appropriate to do so.

We apologize for not including Dis1 in the previous version of the article. We ha his study in this revised version of the manuscript. Furthermore, we have expanded the stu the relevance of Alp14 for the formation of self-assembled meiotic spindles using a thermo-sensitive allele of this protein, *alp14-26*. We have found that meiocytes of the triple mutant *bqt1Δ sad1.2 dis1Δ* can still form self-assembled spindles in a similar percentage compared to *bqt1Δ sad1.2* in the optimal temperature for meiosis progression of 28ºC (Figure 6F). In contrast, *bqt1Δ sad1.2 alp14-26* meiocytes showed a strong reduction of approximately 60% (80% to 30%) in the formation of self-assembled spindles at the semi-permissive temperature of 28˚C (Figure 6F).
Therefore, this new results shows that Alp14 is crucial, even at semi-permissive temperature, for self-assembled spindles, but Dis1 does not play a significant role in our conditions (all data in Figure 6 and Figure S5).

For figure 3 it is important to show co-localisation of Sad1 and Pkl1 and co-localisation of a core SPB marker such as Sid4 and Pkl1 to categorically show that the Pkl1 foci are at the end of microtubule bundles and not SPB remnants.

Thanks for the suggestion, we have now included a set of new experiments with Sid4-mCherry and Sad1.2-mcherry (as SPB core and LINC complex markers, respectively) along with Pkl1-GFP to reinforce our observations (Figure S3). Using these strains, we observed that while the Pkl1 foci are located at the poles, neither SPB nor LINC signals (Sid4 or Sad1, respectively) co-localize with Pkl1.

Lines 230 -232 "…pointing that these dots correspond to γ-tubulin complex association to the uninserted dislodged SPBs." There is a need to show co-staining with an SPB marker here to be able to say this.

Similarly to the previous point, we have repeated the experiments on the association of the gamma-tubulin complex to the non-inserted displaced SPBs using Sid4-mCherry and Sad1- mCherry as markers of the SPB and LINC complex, respectively. Our results have verified that the Alp4-GFP signal is found together with the non-inserted SPBs in the self-assembled spindles setting, while Alp4-GFP is located together with the SPB and the LINC complex in wild-type settings. We have added these experiments to Figure 5 (panels C to F). Thanks for the suggestion.

Line 291 it is important to show and quantitate the differential level of intensity in mixed cultures of the klp6 wild type and klp6 side by side in the same field of view. Then the comparison is truly meaningful. There is always the possibility that differences taken on different sessions on

© 2021. Published by The Company of Biologists under the terms of the Creative Commons Attribution License (https://creativecommons.org/licenses/by/4.0/).

a microscope arise from fluctuations in illumination, capture, or processing. The two lines within the same field of view would maybe differentiated via staining with a cell surface binding lectin, or the expression of a fluorescent marker in one strain but not the other.

Thanks for the comment. Just to clarify that all films have been acquired following strictly the same settings. In any case, we have performed the experiment following the reviewer's suggestion using GFP-tagged F-actin (Life-Act-GFP) to differentiate between wild-type and mutant cells. We choose meiotic cells in the same field and similar meiotic stage. We have added a new Figure S6 with this experiment, the spindle clearly improves in terms of intensity and ability to achieve meiosis II after klp6 deletion in self-assembled spindle settings. We believe that these control experiments support our observations. Thanks again.

Minor points
On line 134 it is stated ", which consists of a dismantling of the whole spindle body (Fig. 2….". The similarity between spindle body and spindle pole body is a bit confusing – the authors may prefer to change the wording – perhaps spindle structure?

Thanks for the suggestion, we agree with the reviewer and have replaced it with "spindle structure" in the revised version.

The gap in the tubulin signal in Figure 2D is not particularly convincing because the entire tubulin structure is comprised of a series of puncta, rather than a linear continuum. It would therefore be good to include some further examples in supplementary data.

We have added a new example in Figure S2. In addition, we have quantified more descriptors, such as spindle length, to support the role of Ase1 in the formation of acentrosomic spindles (see Figure 2F)

Figure 7E – the bars seem to be misaligned in the formation of the pdf document for submission.

We apologize for this problem; although we cannot see this problem, but we have checked the alignments of the bars in this figure. Thanks you.

Reviewer 3 Advance Summary and Potential Significance to Field:
Using live cell microscopy, the authours described the fission yeast double-mutant sad1.2 bqtΔ, where, independent of the SPB, a spindle-like structure can form and can poorly segregate the chromosome at meiosis. They showed that the SPB-independent spindle-like structure has structural similarities to bona fide spindles. Thus, they proposed that the spindle-like structures of fission yeast can be viewed as acentrosomal meiotic spindles, present in other organisms. They then showed that the absence of kinesin-8 in these spindle- like structures enhances their ability to properly segregate chromosomes, suggesting that the same could be true of acentrosomal spindles.

Reviewer 3 Comments for the Author:
I cannot currently recommend publication of the present work in J Cell Sci. There are major concerns:

1) The Abstract effectively states that acentrosomal spindles causes high rate of aneuploidy, which is a strong over-statement. Then the authors claim that the SPB-independent spindle-like structures of fission yeast can be a proxy for studying bonafide acentrosomal spindles and chromosome segregation. This again, is a strong over-statement and simplification.

We apologize if any part of the previous version of the manuscript seemed pretentious about the direct application of our results to mammalian oocytes. We would like to clarify that our work is focused on understanding the molecular mechanisms of self-assembly spindles in S. pombe. As a long-term goal, our idea is to test whether some of the key factors that control these mechanisms in fission yeast could also act in metazoan female meiosis. However, we think that regardless of whether or not our observations are applicable to oocytes, we consider them to be of interest to the field of microtubule and spindle dynamics. We have rewritten the abstract and we

© 2021. Published by The Company of Biologists under the terms of the Creative Commons Attribution License (https://creativecommons.org/licenses/by/4.0/).

have removed sentences as suggested by the reviewer considering them over-statement. We have also rewritten various parts of the introduction and discussion to try to clarify why we think our results are of interest in the field. Morevover, we have performed substantial changes in the paper and added six new figures with a battery of new experiments to reaffirm our conclusions.

2) The description of the fission yeast double-mutant sad1.2 bqtΔ and the SPB-independent spindle-like structures, has been previously reported by the same authors (Pineda-Santaella 2019 Chromosoma). The current work extends the analysis to the function of kinesin-8 - its absence make the spindle-
like structure more efficiency in segregating chromosomes. While this suggests a potential role for kinesin-8 in bona fide acentrosomal spindles, the authours have not validated this.

In line with the previous answer, we have written the text with the idea of focusing on the results in the context of self-assembly in yeast. Although we think that our results will be of interest to be tested in metazoans, it is not the objective of this work. We apologize for the confusion. In the revised version of the article we have reinforced our observations and the independence of the SPB by adding experiments of co-localization of SPB and LINC in the same strains where we analyze the behavior of the gamma tubulin complex and the polarity of the spindle (see the new Figure 5 and Figure S3, respectively please). These data together with previous observations where integral SPB components are not located in self-assembled spindle poles (Figure 2 in Pineda-Santaella & Fernandez-Alvarez, 2019) and the fact that the complete SPB goes to the cytoplasm in bqt1Δ meiocytes (electron microscopy in Figure 1I in Fernandez-Alvarez et al., 2016), in our opinion, they indicate that the self-assembled spindles that we observe are a type of ancestral acentrosomal spindles. As has been observed years ago in xenopus egg extracts, chromosomes can self-assemble a spindle in vitro in the absence of centrosomes. We believe that the possibility of having a system in yeast will be useful to identify the molecular mechanisms behind this type of self-organizing system.

On the other hand, one of our claims is to emphasize that the loss of klp6 improves the robustness of self-assembled spindles. We have reinforced these observations by filming wt and mutant cells in the same field (new Figure S6) and deleting klp5, which reproduces similar results to klp6 although the improvement is much better with klp6 (new Figure 7 and Figure S7). The fact that we have a very significant improvement on self-assembled spindles is, in our opinion, a new and relevant observation. We have eliminated those sentences that induced direct extrapolation to an acentrosomal scenario in female meiosis. We apologise for the confussion.

3) In the SPB-independent spindle-like structures, chromosomes are clearly not properly segregated. However, no markers for kinetochores or centromeres were used in the current study, preventing proper interpretation/conclusion of actual kinetochore-dependent chromosome segregation or passive chromosome separation/fragmentation in these mutants. Again, claiming the SPB-independent spindle-like structure as bonafide acentrosomal spindles is an over-statement.

Thank you for giving us the opportunity to clarify this important point. One of the most crucial controls in our observations was to verify the ability to segregate chromosomes, in other words, to demonstrate that the spindle is functional. For this reason, the behavior of the kinetochores was studied during the extension of the self-assembled spindles. In our previous work, (Pineda-Santaella & Fernandez-Alvarez, 2019 in Figure 5A), where we tagged Mis6 with GFP to visualize the kinetochores. In these experiments, we found that the kinetochores are present in chromosomal masses and separate during the extension of the self-assembled spindles. Now, we have complemented these experiments with snapshots after chromosome segregation in meiosis I and II, which allow us a better signal than in time-lapse films. In Figure S1B, we can see how when chromosomes segregate without SPB separation, kinetochores are present in all masses.

On the other hand, it is important to clarify that when self-assembled spindles are formed, they push the chromosomes but probably because they are weak to complete the segregation to the cell poles, chromosomes return to the center. For this reason, segregation is much clearer in bqt1Δ sad1.2 klp6Δ meiocytes and probably these self-assembled spindles harbor overstable microtubules.

I would be more positive if points 2 and 3 were experimentally addressed.

We think that all the new experiments carried out for the revised version of the article reinforce

© 2021. Published by The Company of Biologists under the terms of the Creative Commons Attribution License (https://creativecommons.org/licenses/by/4.0/).

the existence of self-assembled spindles in fission yeast, their independence from the SPB and their function as bona fide spindles. We apologize again if the first version of the article was so pretentious, we have rewritten the manuscript based on the importance of the self-assembly mechanisms. We believe that some of our observations, such as the role of kinesin-8, could be interesting to explore in the future on female meiosis models, but it is not the main objective of our current work.
Again, we thank all three reviewers for their time, care with the manuscript and suggestions, which we think have led to remarkable improvements in the paper. As a substantial effort have been put into the rewriting of the manuscript and figures, we hope you will now find it suitable for publication in *Journal of Cell Science* and look forward to hearing from you.

We would be glad to respond to any further questions and comments that you may have,
The authors

Second decision letter

MS ID#: JOCES/2020/253799

MS TITLE: Loss of Kinesin-8 improves the robustness of the self-assembled spindle

AUTHORS: Alberto Pineda-Santaella, Nazaret Fernandez-Castillo, Alberto Jimenez-Martin, Maria del Carmen Macias-Cabeza, Angela Sanchez-Gomez, and Alfonso Fernandez-Alvarez

ARTICLE TYPE: Research Article

I am happy to tell you that your manuscript has been accepted for publication in Journal of Cell Science, pending standard ethics checks.

Reviewer 1

Advance summary and potential significance to field

The manuscript has been revised satisfactorily. It reads very much better and includes new data addressing my concerns.

Comments for the author

The manuscript has been revised satisfactorily. It reads very much better and includes new data addressing my concerns.

Reviewer 3

Advance summary and potential significance to field
-

Comments for the author

The authors have addressed all my concerns and suggestions, and therefore I am
in favor of acceptance of the paper.

© 2021. Published by The Company of Biologists under the terms of the Creative Commons Attribution License (https://creativecommons.org/licenses/by/4.0/).
